# Leakage Proof, Flame-Retardant, and Electromagnetic Shield Wood Morphology Genetic Composite Phase Change Materials for Solar Thermal Energy Harvesting

**DOI:** 10.1007/s40820-024-01414-4

**Published:** 2024-05-16

**Authors:** Yuhui Chen, Yang Meng, Jiangyu Zhang, Yuhui Xie, Hua Guo, Mukun He, Xuetao Shi, Yi Mei, Xinxin Sheng, Delong Xie

**Affiliations:** 1 Yunnan Provincial Key Laboratory of Energy Saving in Phosphorus Chemical Engineering and New Phosphorus Materials, The International Joint Laboratory for Sustainable Polymers of Yunnan Province, The Higher Educational Key Laboratory for Phosphorus Chemical Engineering of Yunnan Province, Faculty of Chemical Engineering, Kunming University of Science and Technology, Kunming, 650500 People’s Republic of China; 2 Guangdong Provincial Key Laboratory of Functional Soft Condensed Matter, School of Materials and Energy, Guangdong University of Technology, Guangzhou, 510006 People’s Republic of China; 3 Shaanxi Key Laboratory of Macromolecular Science and Technology, School of Chemistry and Chemical Engineering, Northwestern Polytechnical University, Xi’an, Shaanxi 710072 People’s Republic of China

**Keywords:** Wood PCMs, MXene, Solar thermal storage and conversion, Flame-retardant, Electromagnetic shielding

## Abstract

Phase change materials (PCMs) offer a promising solution to address the challenges posed by intermittency and fluctuations in solar thermal utilization. However, for organic solid–liquid PCMs, issues such as leakage, low thermal conductivity, lack of efficient solar-thermal media, and flammability have constrained their broad applications. Herein, we present an innovative class of versatile composite phase change materials (CPCMs) developed through a facile and environmentally friendly synthesis approach, leveraging the inherent anisotropy and unidirectional porosity of wood aerogel (nanowood) to support polyethylene glycol (PEG). The wood modification process involves the incorporation of phytic acid (PA) and MXene hybrid structure through an evaporation-induced assembly method, which could impart non-leaking PEG filling while concurrently facilitating thermal conduction, light absorption, and flame-retardant. Consequently, the as-prepared wood-based CPCMs showcase enhanced thermal conductivity (0.82 W m^−1^ K^−1^, about 4.6 times than PEG) as well as high latent heat of 135.5 kJ kg^−1^ (91.5% encapsulation) with thermal durability and stability throughout at least 200 heating and cooling cycles, featuring dramatic solar-thermal conversion efficiency up to 98.58%. In addition, with the synergistic effect of phytic acid and MXene, the flame-retardant performance of the CPCMs has been significantly enhanced, showing a self-extinguishing behavior. Moreover, the excellent electromagnetic shielding of 44.45 dB was endowed to the CPCMs, relieving contemporary health hazards associated with electromagnetic waves. Overall, we capitalize on the exquisite wood cell structure with unidirectional transport inherent in the development of multifunctional CPCMs, showcasing the operational principle through a proof-of-concept prototype system.

**Supplementary Information:**

The online version contains supplementary material available at 10.1007/s40820-024-01414-4.

## Highlights

- An innovative class of versatile form-stable composite phase change materials (CPCMs) was fruitfully exploited, featuring MXene/phytic acid hybrid depositing on non-carbonized wood as a robust support.
- The wood-based CPCMs showcase enhanced thermal conductivity of 0.82 W m^−1^ K^−1^ (4.6 times than polyethylene glycol) as well as high latent heat of 135.5 kJ kg^−1^ (91.5% encapsulation) with thermal durability and stability throughout at least 200 heating and cooling cycles.
- The wood-based CPCMs have good solar-thermal-electricity conversion, flame-retardant, and electromagnetic shielding properties.

**Supplementary Information:**

The online version contains supplementary material available at 10.1007/s40820-024-01414-4.

## Graphical abstract

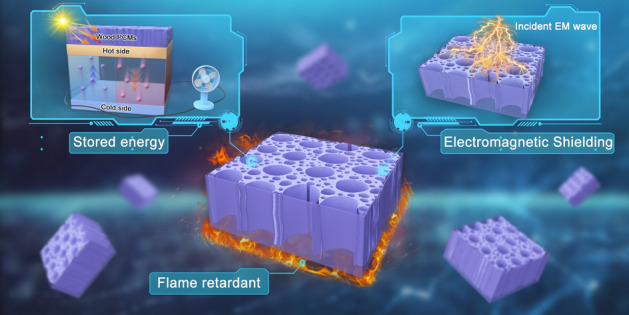

## Introduction

Thermal energy supplementation constitutes the predominant form of global energy consumption, encompassing more than half of the total final energy demand [1, 2]. The escalating challenges posed by the energy crisis and environmental degradation have necessitated a shift in thermal energy acquisition strategies from fossil fuel-based resources to renewable alternatives. Despite this imperative transition, renewable sources like wind energy, tidal energy, and geothermal energy encounter significant drawbacks, including geographical constraints, limited adaptability, and suboptimal efficiency, impeding their widespread adoption [3]. Addressing these limitations requires a comprehensive approach to the management of thermal energy, covering aspects like harvesting, conversion, and storage [4]. In this context, solar thermal conversion technology emerges as a promising avenue for harnessing abundant solar energy, offering notable advantages in terms of conversion efficiency, operational simplicity, and cost-effectiveness [5]. Significantly, phase change materials (PCMs) hold a pivotal role in solar-thermal systems due to their ability to absorb and release substantial thermal energy during phase transitions [6, 7]. This characteristic effectively mitigates challenges associated with solar energy intermittency, dispersion, and efficiency constraints. However, practical implementation of PCM-based solar thermal systems encounters obstacles such as potential leakage, suboptimal thermal storage rates attributable to low thermal conductivity, and a lack of inherent solar thermal conversion capability [8]. Overcoming these challenges is critical to fully unlocking the potential of PCM-based solar thermal systems for advancing sustainable energy solutions.

Achieving shape-stabilization for PCMs through encapsulation techniques such as porous media adsorption [9, 10], microencapsulation [11, 12], and electrospinning [13, 14] within a supporting matrix (*e.g.*, metals, mineral clays, and synthetic polymers) emerges as a promising strategy to effectively mitigate the risk of leakage. In the realm of supporting materials, the three-dimensional (3D) porous scaffold garners significant attention, driven by its straightforward encapsulation process and elevated encapsulation efficiency [15]. In the past decade, materials such as carbon foam [16, 17], synthetic polyurethane foam [18, 19], and aerogels [20, 21] have been extensively employed for the preparation of form-stable composite phase change materials (CPCMs). However, the intricate manufacturing process has resulted in increased production costs as well as the generation of numerous toxic by-products and pollutants, posing a significant challenge to the principles of sustainable development. Therefore, there is a compelling need for the development of 3D porous scaffolds that are both effective and sustainable, featuring ease of manufacturing and ensuring environmental friendliness. Fortunately, the exquisite structures found in biological organisms have inspired the development of alternative synthetic encapsulation matrices [22]. Derived from natural structures, biobased materials can be obtained through a facile “top-down approach”, eliminating the need for complex construction processes [23–25]. As for wood, a prime example of nature’s intricate design, featuring meticulously aligned structures like hollow vessels, tracheid elements, and membranes such as pits designed for efficient water and ion transport [26]. The hierarchical porosity inherent in wood, extending from the macroscale to the nanoscale, positions it as a highly promising functional material [27, 28]. Beyond its well-known roles in liquid absorption and fluid filtration [29, 30], wood emerges as a compelling candidate for encapsulating PCMs. Liu et al. [31] achieved encapsulation efficiencies of 83.9%, 84.0%, and 74.1% for myristic acid, paraffin, and polyethylene glycol, respectively, in balsa wood. Yang et al. [32] encapsulated 1-tetradecanol in basswood with an efficiency of 59.94% and a phase change enthalpy of 124.6 J g^−1^. A frequently utilized procedure in these studies involves the removal of lignin, which significantly diminishes light absorption across the visible and ultraviolet light spectra. The weakened photo-absorption capabilities of both wood, particularly after delignification, and the PCM collectively contribute to a reduced efficiency in converting solar irradiation. Consequently, these limitations curtail the broader applications of wood-based CPCMs in the domain of solar to thermal energy management.

Recently, the integration of Fe_3_O_4_ [33] and carbon quantum dots [34, 35] directly into PCMs, followed by encapsulation within a wooden scaffold, has demonstrated notable improvements in both photothermal conversion efficiency and thermal conductivity. However, the direct addition of these components does not facilitate effective bonding between fillers, and there is also a lack of proper adhesion to the framework. Consequently, during prolonged usage, the fillers are prone to agglomeration and failure under the influence of gravitational forces. It is reported that utilizing surface engineering techniques to foster the growth of functional two-dimensional (2D) nanofillers on the surface of the wood-derived scaffold represents a viable approach to address the aforementioned challenges [36, 37]. For instance, MXene, a novel 2D stacked lamellar material constituting transition metal carbides and carbonitrides, is obtained through the etching and exfoliation of MAX phases [38], showcasing exceptional capabilities in photothermal conversion [39], thermal conductivity [40], and optical application [41]. In addition, the surface of MXene is enriched with plentiful active functional groups, such as ‒OH, ‒COOH, and ‒F, facilitating the deposition on the hydrophilic surface of the substrate, particularly for delignified wood [42–44]. As reported, MXene can be effectively anchored to the surface of wood through hydrogen bonding and could be used in the field of electromagnetic shielding [45], flame retardancy [46], volatile organic compounds (VOCs) detection [47], solar steam generation [48], light-driven actuator [49], electrode [50], and environmental purification [51]. However, there has been limited reporting on the application of MXene modified wood in the field of solar to thermal energy conversion and storage. Here, the bonding between MXene and PCMs, as well as their collaborative flame-retardant and electromagnetic shielding properties, represents a crucial area for further exploration and research.

In this study, an innovative class of versatile form-stable CPCMs was fruitfully exploited, featuring MXene (Ti_3_C_2_T_x_) depositing on non-carbonized wood as a robust support. The fabrication process involved selective wood delignification via boiling in an acidic NaClO_2_ solution, followed by phytic acid (PA) and MXene co-adornment. Subsequently, polyethylene glycol (PEG) as the main PCM was encapsulated through vacuum impregnation, ultimately resulting in the formation of CPCMs that showcase exceptional solar-thermal conversion efficiency, highly efficient electromagnetic interference shielding, and outstanding flame-retardancy. The lightweight nanowood derived from the removal of lignin that retains honeycomb-like oriented porous structures, namely wood aerogel, with their robust surface tension and remarkable capillary force, and the MXene/PA hybrid surface structure with functional groups capable of forming hydrogen bonds, collaboratively played a pivotal role in effectively encapsulating PEG molecules. This encapsulation mechanism proved to be instrumental in preventing leakage during thermal storage processes. A comprehensive evaluation, encompassing chemical structure, crystalline state, microscopic morphology, encapsulation capability, phase change performance, thermal repeatability, fire-retardant behavior, solar–thermal–electricity conversion, and electromagnetic interference shielding effectiveness, was conducted to provide a nuanced understanding of the overall properties of the CPCMs. As anticipated, the multi-faceted approach via facile MXene and PA hybrid wood modification promotes the multifunctionalization of the as-prepared form-stable CPCMs, making them promising candidates for diverse applications.

## Experimental Section

### Fabrication of Wood Aerogel and MXene/PA Hybrid Modification

Balsa wood timber was initially cut into samples with dimensions of 10 mm × 10 mm × 3 mm. A certain number of bulk wood samples were selected and placed in a 1 wt% sodium chlorite/acetic acid solution, maintaining a pH of 4.6 for boiling treatment. After approximately 18 h, the color of the wood blocks changed from light yellow to white. The delignification treatment was stopped, and the samples were then rinsed with deionized water 3–5 times. After freeze-drying, the wood aerogel was obtained and denoted as DW.

DW underwent a hybrid modification process with MXene and PA, employing the evaporation-induced assembly method. Initially, the MXene dispersion was diluted to 2 mg mL^−1^ using deionized water, and a specific mass of PA was added to form an MXene/PA hybrid dispersion known as MP. Subsequently, MP dispersion was meticulously added dropwise to DW using a pipette. The volume of added MP was controlled based on the MXene mass fraction in DW, namely 2%, 5%, 7%, and 10%. The ensuing hybrid-modified wood was dried in an oven at 60 °C, resulting in distinctive wrinkled structures. Subsequently, deionized water was added dropwise to allow the modified wood blocks to swell to their original size. Afterward, freeze-drying for 24 h resulted in the modified wood aerogel. Depending on the MXene content, these were labeled as MPxDW, where *X* = 2, 5, 7, and 10, representing MXene mass fractions in the wood aerogel framework of 2%, 5%, 7%, and 10%, respectively.

### Impregnation with PEG for the Preparation of CPCMs

The prepared MPxDW samples were immersed in PEG using a vacuum oven at 80 °C, 0.1 MPa according to our earlier studies. After 10 h, the wood-based composite phase change materials were all extracted, and based on the different encapsulation frameworks, they were labeled as MP2DWP, MP5DWP, MP7DWP, and MP10DWP, respectively. Subsequently, a leakage experiment was conducted to determine the encapsulation capacity of different wood frameworks in the CPCMs. Specifically, all the aforementioned PEG-filled composite phase change materials were placed on filter paper and placed in an 80 °C. The weight was measured every 15 min until the mass no longer changed, thus obtaining completely encapsulated MPDWPs.

Other comprehensive details related to the experimental procedures, encompassing the sources and types of materials, preparation of MXene aqueous dispersion, delignification and hybrid modification of wood, fabrication of CPCMs, structural characterization, and the evaluation of solar to electricity conversion and electromagnetic shielding performance, are all demonstrated in Supporting Information.

## Results and Discussion

### Balsa-Derived Framework and MXene/PA Hybrid Modification

**Fig. 1:**
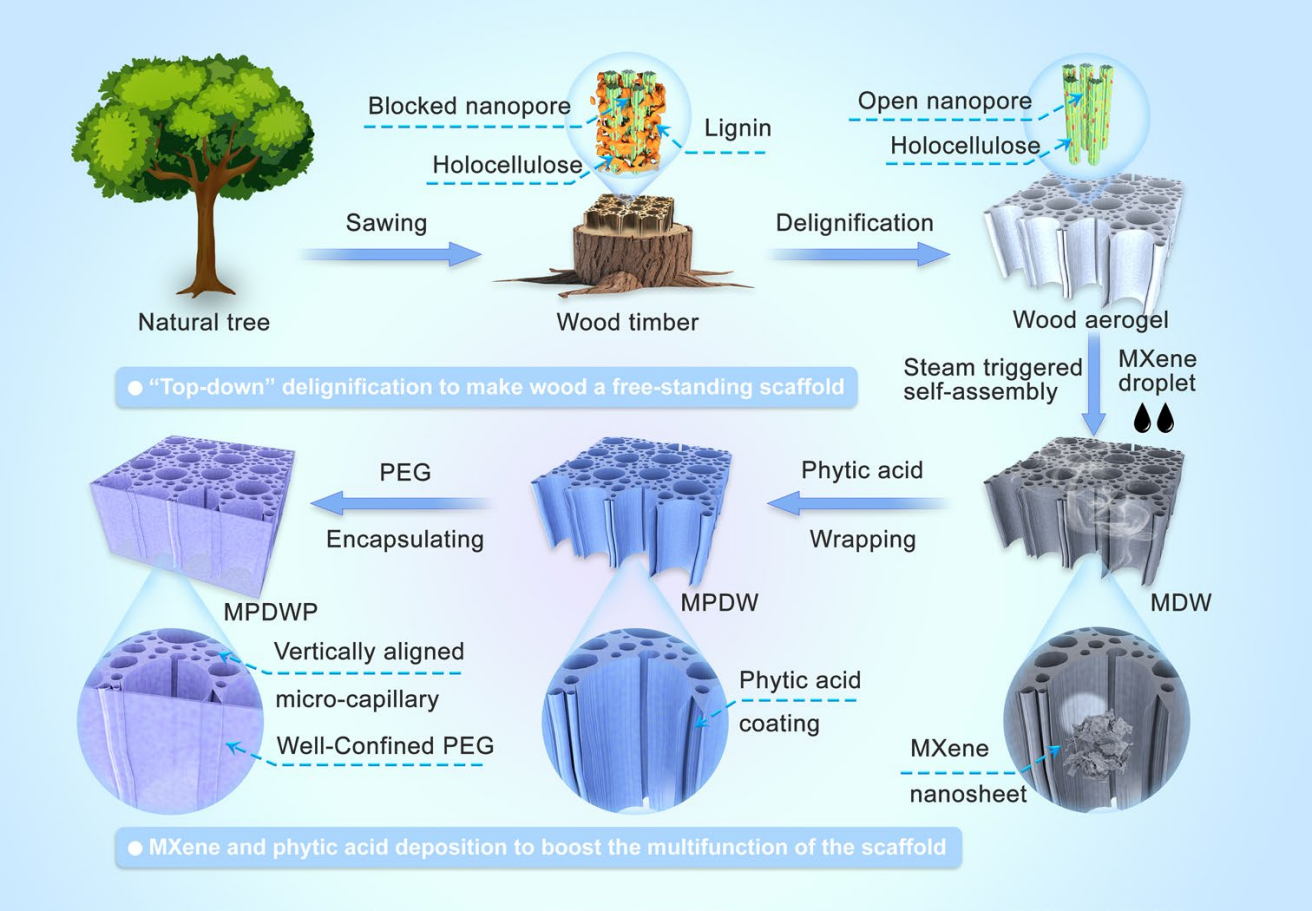
Schematic diagram of the preparation of composite phase change materials supported by phytic acid and MXene-decorated wood aerogel

The fabrication process of wood-based 3D frameworks with unidirectional pore structures as well as designed functionalized composite phase change materials is illustrated in Fig. 1. As the lightest natural wood globally, balsa (*Ochroma pyramidale*) boasts remarkably rapid growth, featuring the content of hollow tracheid tissues as high as 90%. Tracheids constitute a vertically arranged honeycomb-like capillary structure with a diameter of approximately 10 μm (Fig. S1a1), rendering balsa a highly promising encapsulation matrix for the fabrication of form-stable phase change materials. However, fluctuations in nutrition and environmental conditions of balsa growth led to the formation of obstructive membrane structures and sealed pits (Fig. S1a2), which always limit the PCM effectively penetrating and encapsulating. Li et al. [52] also confirm that unmodified wood exhibits only a lower level of encapsulation efficiency. Hence, the raw balsa timber undergoes treatment in an acidic sodium chlorite solution, facilitating the substantial removal of lignin while preserving hemicellulose [37]. This facile delignification process disrupts the inherent closed structure, enhancing permeability and exposes more ‒OH groups on tracheid surfaces, facilitating subsequent functional modifications. Meanwhile, the retention of hemicellulose prevents significant deformation under external forces, thereby augmenting the structural stability of the framework. As anticipated, the mild delignification process has not altered the overall upright pore structure of balsa wood (Fig. S1b1). Instead, more open pore structures and gaps have appeared between the saturated cell wall layers (Fig. S1b2, b3). The modified balsa retains only carbohydrate components, resulting in an approximately 40% reduction in density (Table S1), rendering balsa to be a cellulosic aerogel with a well-defined unidirectional pore structure. Compared to synthetic encapsulation matrices with higher density or disordered pores (*e.g.*, polyurethane), the as-obtained wood aerogel will demonstrate significantly improved encapsulation efficiency and stability for PCMs.

**Fig. 2:**
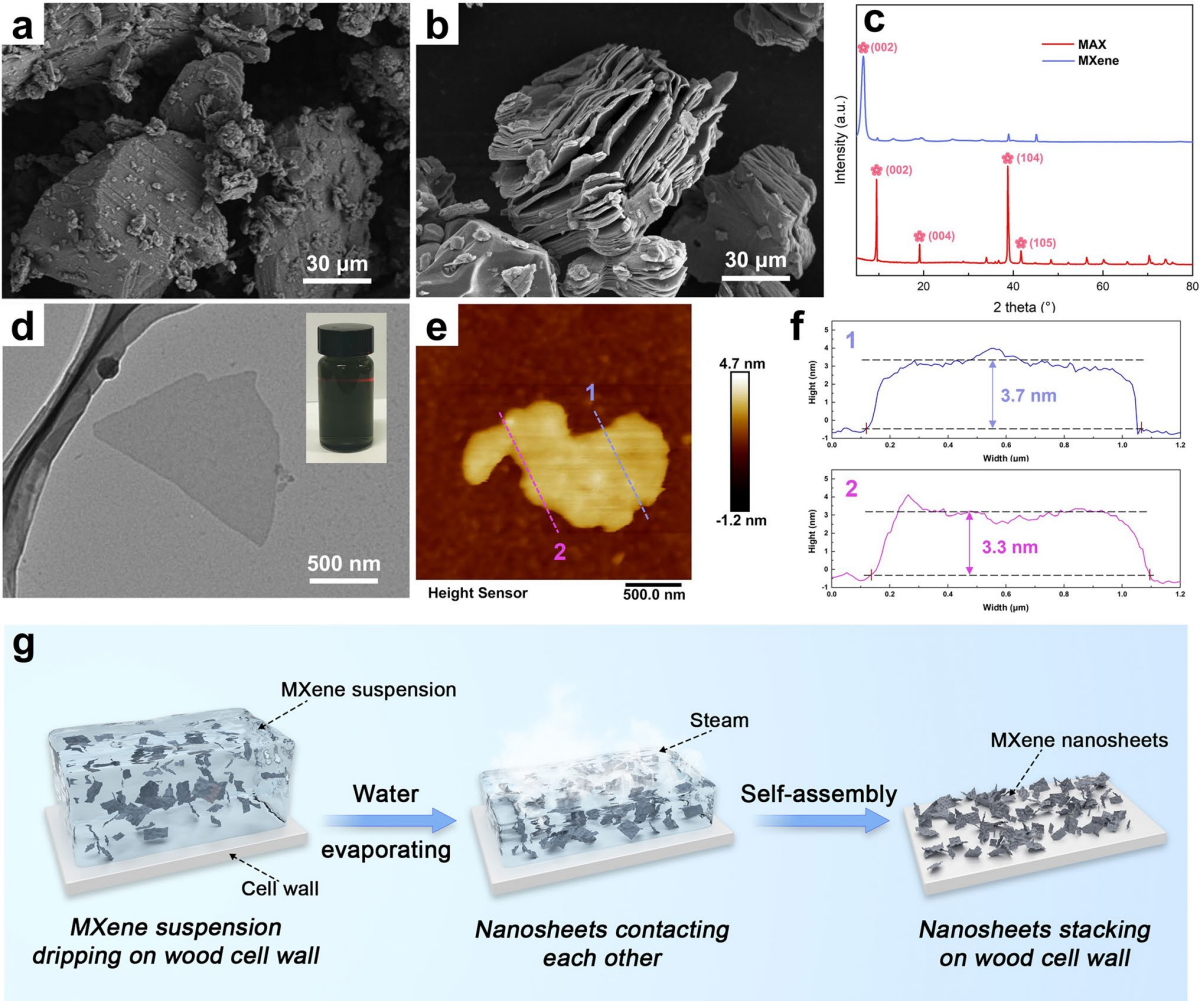
SEM micrographs of **a** MAX and **b** multilayered MXene. **c** XRD patterns of MAX and MXene. **d** TEM images of MXene and optical image in the thumbnail. **e** AFM images of MXene. **f** AFM corresponding height profiles of MXene. **g** Illustration to show the self-assembly process of MXene nanosheets triggered by fast-evaporating water in MXene suspension

MXene has been meticulously selected as a 2D nanofiller with the explicit goal of functionalizing nanowood to ameliorate its inherent deficiencies (*e.g.,* low thermal conductivity, flammability, and a lack of photothermal conversion performance) for the preparation of versatile form-stable CPCMs. The bulk MAX (Ti_3_AlC_2_) phase with a densely stacked structure serves as the starting material (Fig. 2a), and an accordion-like loose MXene (Ti_3_C_2_) layer (Fig. 2b) is formed by selectively etching its internal Al layers with a LiF-HCl solution. The success of the aforementioned etching process is further evidenced by changes in the crystalline structure. As shown in the spectra of XRD (Fig. 2c), the characteristic diffraction peak (104) located at 39° in correlation to Al element as well as the other diffraction peaks including 004 and 105 in MAX undergoes a significant reduction and even disappearance following the transformation of MXene. Meanwhile, the shift of the characteristic peak at 002 from 9.6 to 7.3° signifies an expanded d-spacing from 0.94 to 1.18 nm in MXene after Al layer removal, resulting in an overall increase in the interlayer distance, which is in coincidence with the changes on microscopic morphology (Fig. 2a, b). Subsequent ultrasonication enables the delamination of MXene, yielding a dispersion of single-layer or few-layer MXene that exhibits pronounced Tyndall scattering (Fig. 2d). This attests to the superior dispersibility of MXene in water, owing to its abundant hydrophilic functional groups (*e.g.,* ‒OH, ‒COOH, and ‒F) [53, 54]. TEM morphology in Fig. 2d reveals that the exfoliation of multilayer MXene, deprived of van der Waals forces, adopts an ultra-thin nanosheet structure. AFM measurements further confirm that the width of the aforementioned ultra-thin nanosheets falls within the range of 0.4–1.0 μm, while the thickness ranges from 3.3 to 3.7 nm (Fig. 2e, f), which are consistent with the findings by Cao et al. [55]. Collectively, a facile process involving delamination and ultrasonication has successfully yielded single-layer or few-layer MXene nanosheets, awarding a promising avenue for subsequent functional amelioration of nanowood. As illustrated in Fig. 2g, the suspension, comprising uniformly dispersed MXene nanosheets, could permeate the entire lumens within the nanowood. Subsequent rapid water evaporation will facilitate the contact between MXene nanosheets to form hydrogen bonds, which ensures the spontaneous layer-by-layer deposition of MXene nanosheets onto the nanowood surface, thus to establish a continuous MXene-based functional structure.

**Fig. 3:**
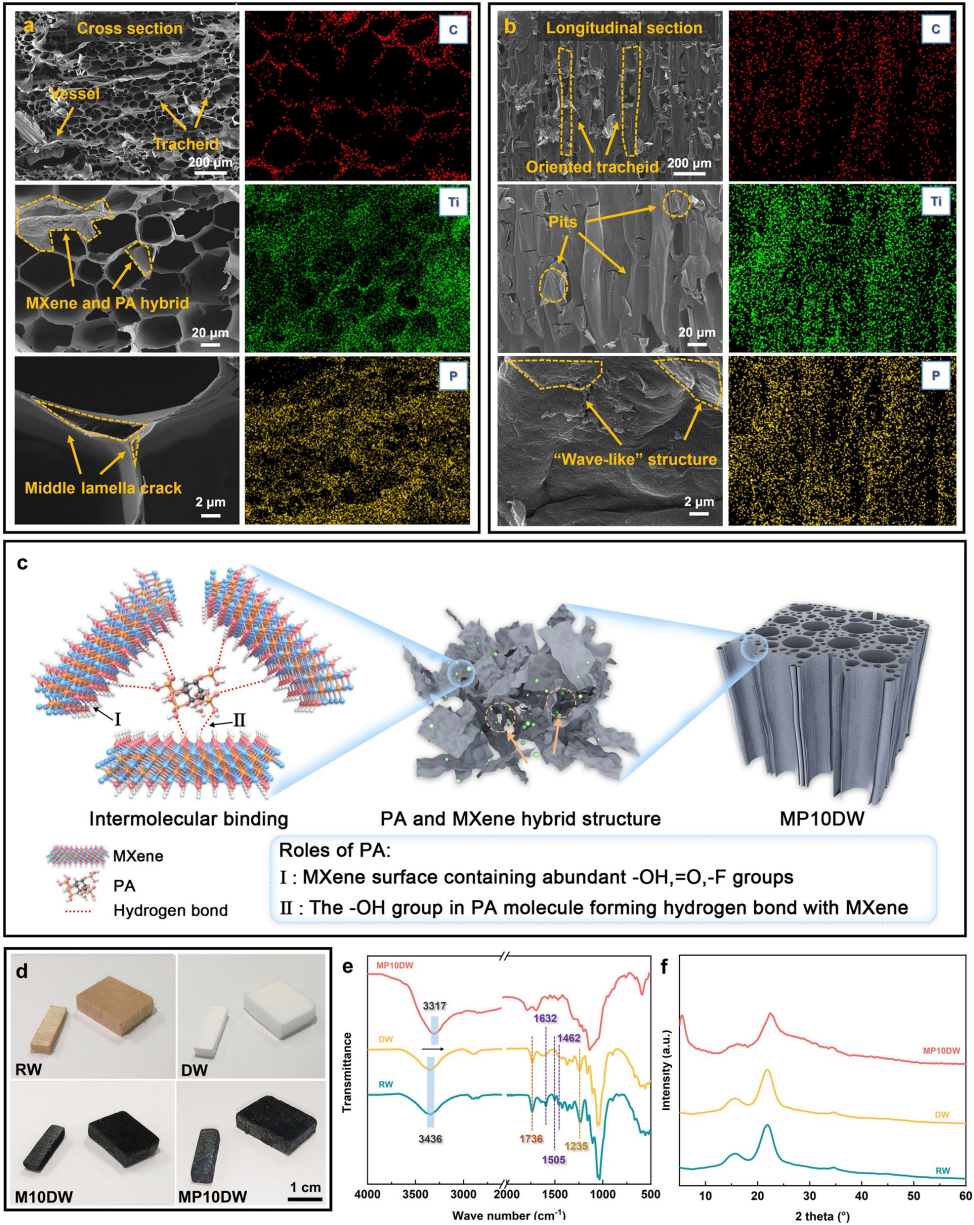
Field-emission scanning electron microscope and energy-dispersive spectroscopy images of wood aerogel-loaded MXene and PA (MP10DW) in **a** cross view, **b** longitudinal view. **c** Illustration to show the roles of PA in forming stable MXene hybrid structure. **d** Photographs of RW, DW, M10DW, and MP10DW. **e** FTIR and **f** XRD of RW, DW, and MP10DW

Despite the considerable body of study on documenting the utilization of MXene nanosheets for the modification of wood-based materials, relying exclusively on the limited hydroxyl and carboxyl groups on the MXene surface to form bonds through weak interactions with the wood cell wall often proves to be unreliable, which are not conducive to long-term applications [56]. Therefore, we introduced phytic acid, which possesses a polyhydroxyl structure, into the adhesion of MXene on the nanowood lumen surface, forming a hybrid structure to enhance hydrogen bonding interactions between the constituents (Fig. 3c). As anticipated, the co-modification process of PA and MXene, illustrated in Fig. 3a, b, preserves the original pore structure of the nanowood. The oriented arrangement of tracheids, open pits, and defects between cell walls remains intact. This can be attributed to the mild modification process of MXene and PA, which occurs at room temperature without the involvement of additional toxic solvents. In addition, some “wave-like” structures are observed on both cross-sectional and longitudinal views, which may result from the stacking of hybrid structures formed by MXene and PA. EDS mapping further reveals that C elements predominantly depict the framework structure of the cell walls both in axial and longitudinal directions. Concurrently, Ti elements from MXene and P elements from PA also exhibit a well-uniform distribution. All these findings collectively indicate the successful formation of a MXene/PA hybrid structure on the nanowood surface.

The alterations in the surface chemical structure of the composite material, arising from delignification and deposition of the hybrid MXene/PA structure, were further elucidated by FTIR, in Fig. 3e. For the raw balsa wood, distinct absorption peaks appeared at approximately 1736 and 1235 cm^−1^, corresponding to the stretching vibrations of carbonyl groups (C–O) and ester groups (–CO–OR), respectively [57]. Both are indicative of hemicelluloses, specifically amorphous polysaccharides. Also, faint peaks detected at 1590, 1505, and 1462 cm^−1^ signify aromatic skeletal vibrations, attributing the presence of lignin in the pristine balsa wood [37]. Following the delignification, the selective removal of lignin is evidenced by the disappearance of characteristic lignin peaks, while the peaks associated with hemicelluloses remain preserved. This process also causes the balsa wood to change from yellowish to white (Fig. 3d), attributable to the disappearance of chromophoric groups composed of lignin. In addition, the changes in chemical composition including cellulose, hemicellulose, and lignin of raw balsa wood (RW) and DW are depicted in Table S1. As shown in Fig. 3d, subsequent MXene modification results in the blackening of MP10DW, which is evident not only on the surface but extends to every internal channel. The following incorporation of phytic acid did not induce noticeable alterations of MP10DW in the observed black coloration, indicating that phytic acid primarily functions in structural enhancement without impacting the potential photothermal conversion performance. Furthermore, there is no significant change observed in the FTIR of MP10DW, except for a discernible shift in the –OH characteristic peaks from 3436 to 3417 cm^−1^, substantiating the formation of hydrogen bonds, which is also confirmed by the XPS measurements (Fig. S2) [49]. XRD spectra in Fig. 3f also reveal characteristic diffraction peaks located at 2*θ* = 16.5°, 22.5°, and 34°, corresponding to the (101), (020), and (040) crystal planes of cellulose I, respectively [58]. These peaks persist in RW, DW, and MP10DW with minimal variation, underscoring that the delignification and hybrid modification processes do not disrupt the crystalline structure of cellulose. Notably, the characteristic peak of MXene at 2*θ* = 7.4° emerges in MP10DW. These findings affirm that the MXene/PA co-modification is primarily a physical interaction, safeguarding the structural integrity of both the nanowood framework and MXene, which lays the foundation for subsequent synergistic functional enhancements.

### CPCMs Morphology and Encapsulation Capability

**Fig. 4:**
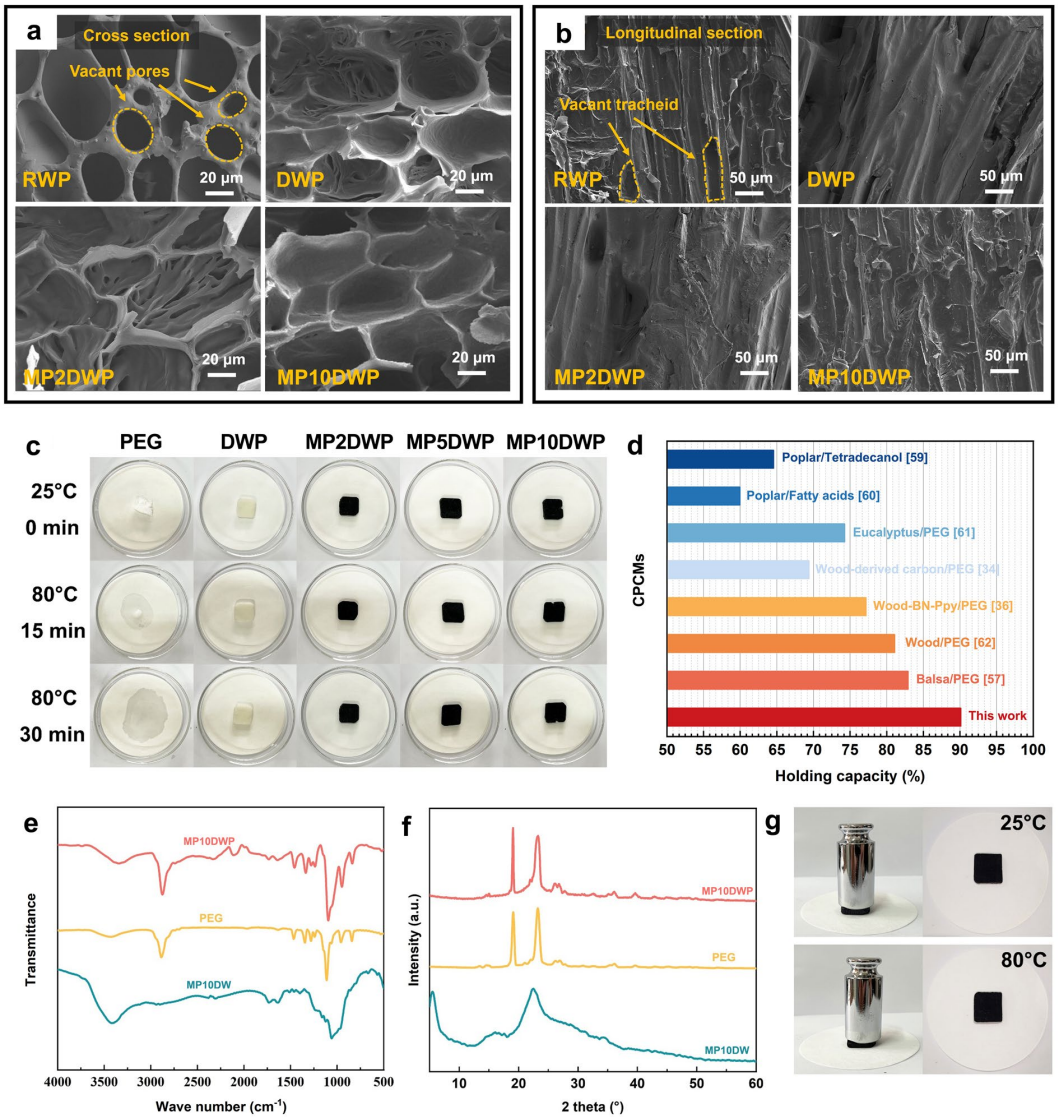
Field-emission scanning electron microscope of RWP, DWP, MP2DWP, and MP10DWP in **a** cross view and **b** longitudinal view. **c** Leak-proof performance evaluation of PEG and MPDWPs at 25 and 80 °C. **d** Encapsulation capability for the wood-derived substrates prepared in this work compared to other porous materials from literature [34, 36, 57, 59–62]. **e** FTIR and **f** XRD of MP10DW, PEG, and MP10DWP. **g** Photographs of the mechanical test at 25 and 80 °C

PEG was incorporated into the modified balsa wood using a vacuum impregnation technique, yielding form-stable composite phase change materials. In SEM images (Fig. 4a, b), it is evident that raw balsa wood used for encapsulating PCMs, RWP, displays numerous unfilled voids, potentially attributed to inherent defects in the natural wood structure. However, following the lignin removal process, the voids can be completely filled in DWP, indicating the enhanced effectiveness of lignin removal in facilitating encapsulation. Importantly, as for MP2DWP and MP10DWP, the MXene and PA hybrid modification does not adversely impact the encapsulation capacity, as the voids remain filled even after the increase of MXene contents. This observation can be attributed to the fact that the hybrid modification did not cause pore blockage in the nanowood scaffold, while the exposure of more hydrophilic groups (*e.g.,* –OH, –COOH, and –F) enhanced the hydrogen bonding interactions with PEG [55]. In addition, the shape stability of CPCMs is crucial for practical applications. As depicted in Fig. 4c, all samples are subjected to constant temperature conditions in a vacuum oven at 80 °C. Clearly, pure PEG exhibits leakage after 15 min of heating. Conversely, DWP and all MPDWPs maintain their intact shape without any leakage. Even after 80 min, pure PEG completely undergoes amorphous melting, while the MPDWPs maintain their original shape macroscopically, showcasing the beneficial effects of the rigid 3D supporting framework and strong capillary forces provided by MPDWs, facilitating PEG absorption. Furthermore, as shown in Fig. 4g, the MP10DWP is capable of supporting a 100 g weight, even at a temperature of 80 °C, without any leakage, which indicates that the oriented nanowood scaffold exhibits significant strength in the vertical direction, enabling it to resist external forces in the surrounding environment and thereby enhancing structural stability. Notably, the encapsulation mass ratios for MP10DWP samples in our study are significantly higher than those reported for wood-based PCMs in the literature (Fig. 4d). Furthermore, the characteristic peaks observed in FTIR analysis (Fig. 4e) and XRD analysis (Fig. 4f) for MP10DWP are similar to those of PEG. These results indicate that the chemical and crystal structure of PEG remains unchanged in MPDWPs, confirming that the porous structure of MPDWs has no impact on the characteristic groups and crystallinity of PEG. Therefore, the phase change properties of MPDWPs are expected to be unaffected by the composite structure and remain consistent with those of pure PEG. Collectively, these findings validate the excellent loading capability of wood aerogels decorated with MXene and PA for PEG, while highlighting the favorable shape stability and reliability of MPDWPs.

### Thermal Properties and Phase Change Behavior of the Balsa-Derived CPCMs

**Fig. 5:**
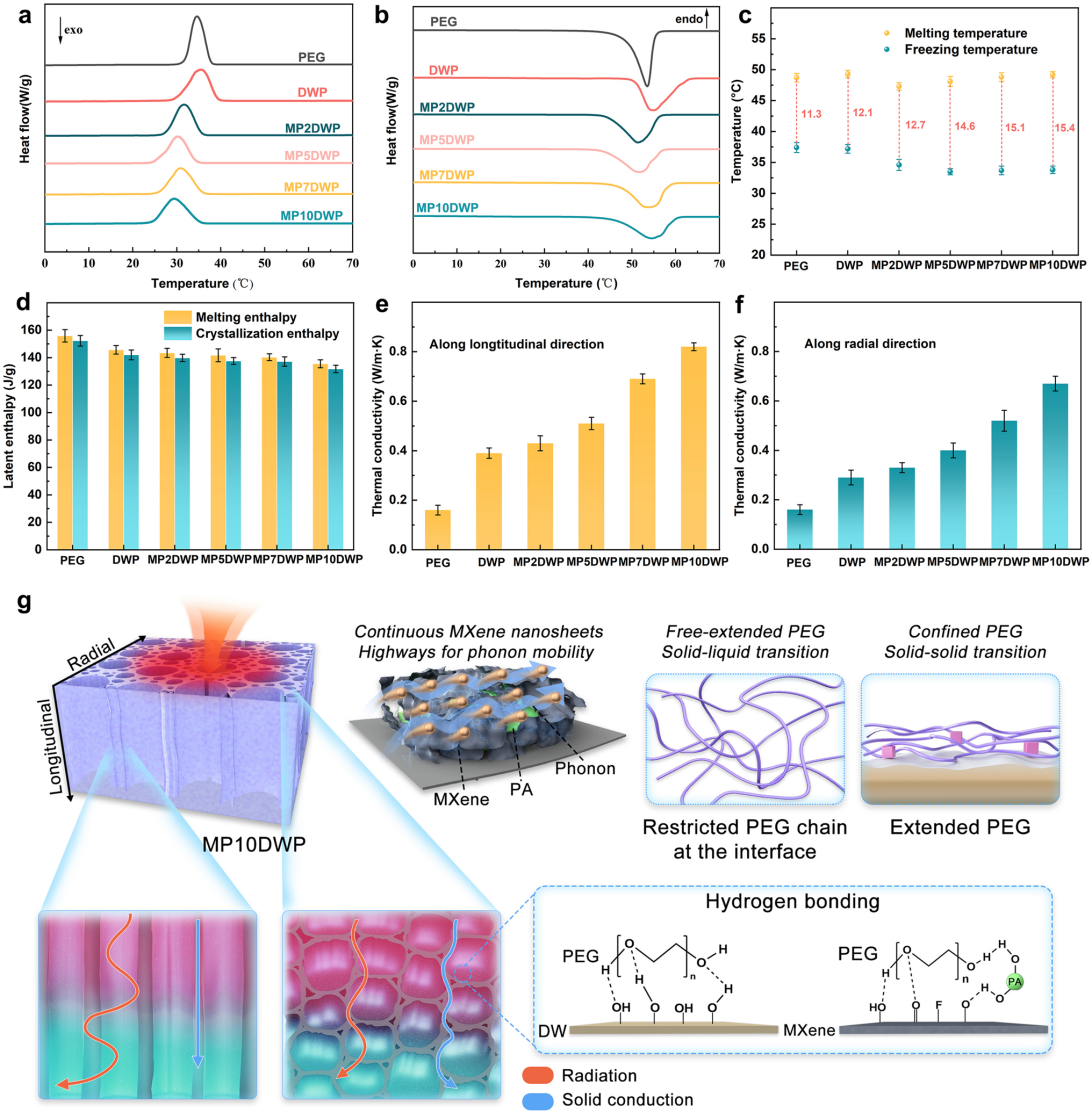
DSC thermograms for MPDWPs upon **a** cooling and **b** heating. **c** Degree of supercooling of pure PEG, DWP, and MPDWPs, respectively. **d** Enthalpy values of MPDWPs. **e** Longitudinal and** f** radial thermal conductivity of pure PEG, DWP, and MPDWPs, respectively. **g** Possible mechanism of enhanced thermal conductivity and molecule interactions in MP10DWP and the PEG chain motion during phase transition

Practical applications of thermal energy management demand CPCMs with a robust trifecta: a substantial latent heat storage capacity, an apt phase transition temperature, and durable 3D structures. To unravel the thermal intricacies and phase transition behaviors, differential scanning calorimetry (DSC) was employed. In Fig. 5a, b, DSC curves delineate the endothermic and exothermic cycles for both pure PEG and the CPCMs (*e.g.,* DWP, MP2DWP, MP5DWP, MP7DWP, and MP10DWP). Key insights from DSC curves, encompassing parameters such as melting point (*T*_*m*_), fusion enthalpy (Δ*H*_*m*_), freezing point (*T*_*c*_), and solidification enthalpy (Δ*H*_*c*_), are succinctly presented in Table S2. Specifically, the energy storage efficiency (*F*) is calculated using the following formula (Eq. 1) to assess the influence of the encapsulation framework on the overall phase change performance [33].

1 $$F = \frac{{\Delta \,H_{{m, {\text{CPCMs}}}} }}{{\Delta \,H_{{m,{\text{PEG}}}} }} \times 100\%$$

The terms *ΔH*_*m,* CPCMs_ and *ΔH*_*m, *PEG_ signify the measured phase change enthalpy for the CPCMs and PEG, respectively. As depicted in Fig. 5a, b, the DSC curves of the CPCMs closely resemble those of pure PEG, featuring distinct endothermic and exothermic peaks indicative of solid–liquid and liquid–solid phase transitions. This observation suggests successful PEG encapsulation within the nanowood scaffolds, maintaining its inherent phase change properties without undergoing chemical interactions. As common sense, optimal thermal energy storage capacity, with a phase transition enthalpy closely aligned with the neat PCM, is desirable for broad applications in thermal storage. For pure PEG, the phase transition enthalpies measure 155.9 and 152.3 kJ kg^−1^, respectively. In the case of DWP, the close enthalpy comparable to that of pure PEG (145.7 and 142.0 kJ kg^−1^) is primarily due to the interconnected pore structure and lightweight facilitated by DW. Notably, the phase transition enthalpy values for MPDWPs derived from hybrid-modified nanowood diminution as MXene deposition contents are increased (Fig. 5d). Particularly, the fusion enthalpy and solidification enthalpy of CPCMs exhibit variations in the ranges of 143.5–135.5 kJ kg^−1^ and 139.8–131.8 kJ kg^−1^, respectively, with the effective energy storage coefficient consistently exceeding 85%. This indicates that the influence of hybrid functional modification on the encapsulation performance of the framework is relatively minimal, and the acceptable sacrifice in enthalpy values is evident. Here, the introduction of the expected value *E* (*F*/*I*) serves as a metric to gauge the degree of influence the encapsulation framework has on phase change enthalpy values [36]. The *E* value of DWP is equivalent to 0.98, while, for MP2DWP, MP5DWP, MP7DWP, and MP10DWP, the calculated *F*/*I* amounts to 0.97 and 0.95, respectively, indicating that the deposition of MXene and PA hybrid structures on the surface of nanowood exhibits an unfavorable impact on phase change behavior of encapsulated PEG. Furthermore, the introduction of MXene as a thermal conductive filler has significantly enhanced the thermal conductivity of the CPCMs. This trend is evident both along the longitudinal and radial directions, displaying an increasing tendency with the rising MXene contents.

The illustration in Fig. 5g outlines the thermal conductivity enhancement in CPCMs and the proposed mechanism for phase change performance fluctuations. Pure PEG exhibits inherently low thermal conductivity (0.18 W m^−1^ K^−1^), a common limitation of organic PCMs. When encapsulated in nanowood, the oriented framework induces anisotropic thermal conductivity. Along the longitudinal direction, upright axial tracheid cell walls create directed thermal pathways, facilitating rapid heat conduction (Fig. 5e). However, for DWP, due to the inherently low thermal conductivity of nanowood, the increase is only by a factor of 2.2 times. In the radial direction (Fig. 5f), heat conduction involves repetitions through thin cell walls to PEG and back, resulting in a less significant increase, merely by a factor of 1.6 times. These findings align with Yang et al. [33]’s study. The introduction of MXene as a thermal conductive filler substantially boosts the CPCMs’ thermal conductivity. Specifically, with 10% MXene content, the thermal conductivity increases significantly, reaching 0.82 W m^−1^ K^−1^ (approximately 4.6 times higher than that of pure PEG) in the longitudinal direction, comparable to our earlier findings and surpassing other wood-based materials (Table S3). This enhancement could be attributed to the stable continuous structure formed by a high MXene content on the wood cell wall surface, facilitating photon transmission and an overall increase in thermal conductivity [44, 63, 64]. After delignification, the exposed nanowood framework reveals more –OH groups, forming hydrogen bonds with PEG molecules at the interface to prevent leakage. However, PEG molecules anchored by hydrogen bonding lose their free movement due to confinement effects, altering the solid–liquid phase change to solid–solid, resulting in a decrease in the expected enthalpy value (*E*, 95%) [65]. MXene/PA hybrid structures at the interface introduce more hydrophilic groups (*e.g.*, –OH, –COOH, and –F), forming additional hydrogen bonds with PEG molecules. The increased PEG anchoring at the interface leads to an expected value decrease from 0.97 to 0.95 for MPDWPs. Fusion and solidification points are critical for CPCMs. A smaller difference, *i.e.*, less supercooling, is preferred. The change in melting and freezing points relates to thermal conductivity and interface confinement effects. Better thermal conductivity lowers melting and freezing points. For PEG and CPCMs, fusion and solidification points initially decrease and then increase, indicating an initial improvement in thermal conductivity (Fig. 5c). However, as MXene and PA content rises, interface confinement effects dominate, causing melting and boiling points to rise again. This results in a continuous increase in supercooling from 12.1 to 15.4 °C. Overall, an expected value greater than 90%, with supercooling less than 20 °C, is considered acceptable for practical CPCM applications [66, 67].

### Thermal Reliability and Repeatability of Balsa-Derived CPCMs

**Fig. 6:**
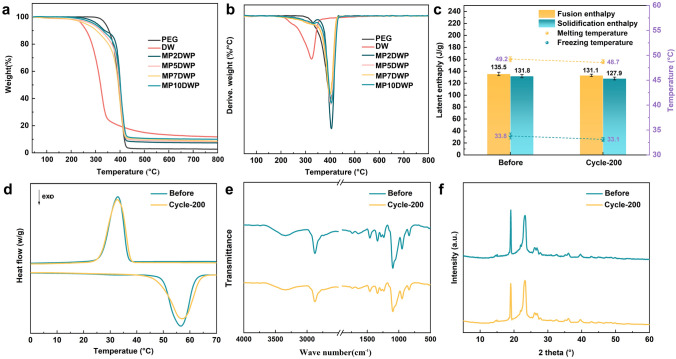
**a** TGA and **b** DTG of DW, PEG, and MPDWPs. Stability and recyclability of MP10DWP before and after 200 consecutive cycles of heating and cooling as observed by **c** enthalpy values, **d** DSC, **e** FTIR, and **f** XRD measurements

The thermal reliability of CPCMs is a pivotal factor in ensuring their stable application. Thermal gravimetric (TG) and derivative thermogravimetric (DTG) analyses were conducted under a nitrogen atmosphere for pure PEG, unmodified nanowood framework, and CPCMs, as depicted in Fig. 6a, b with corresponding parameters recorded in Table S4. Compared to neat PEG, the nanowood framework exhibits an earlier onset of thermal decomposition at 201.3 °C, attributed to the poor thermal stability of the cellulosic components. In the case of PEG encapsulated in hybrid-modified nanowood, MPDWPs display thermal decomposition curves similar to pure PEG, undergoing one stage within a specific temperature range from 210.5 to 425.7 °C. This observation can be attributed to the encapsulation rate of PEG exceeding 90% and the addition of a hybrid structure with MXene and PA that facilitates the carbonization process during decomposition. As expected, the aforementioned hypotheses are substantiated by the residual carbon values of MPDWPs. With an increase in MXene content, there is an observable upward trend in residual carbon values, notably reaching 7.3% at 10.1% MXene content. Considering practical applications of CPCMs, particularly in solar-to-thermal conversion and storage, all MPDWPs exhibit barely thermal degradation below 200 °C, implying that the MPWPs demonstrate acceptable thermal stability for their intended applications.

The assessment of repeatability stands as another pivotal criterion in gauging the practical longevity of CPCMs. To thoroughly evaluate this aspect, rigorous 200 heating–cooling cycle tests were conducted across the 25–80 °C temperature range to gauge the reusability of MP10DWP. Remarkably, the DSC curves of MP10DWP after 200 thermal cycles closely mirror those of the original one (Fig. 6d). Furthermore, as shown in Fig. 6c, the computed loss of fusion and solidification enthalpy after 200 thermal cycles are a mere decrease from 135.5 to 131.1 kJ kg^−1^ and 131.8 to 127.9 kJ kg^−1^, respectively. Concerning the phase change temperatures, there is also subtle variation observed. Specifically, the melting point has marginally decreased from 49.2 to 48.7 °C, and correspondingly, the freezing point has transitioned from 33.8 to 33.1 °C, affirming that MP10DWP upholds their elevated phase change performance throughout protracted cyclic testing. Moreover, following 200 thermal cycle experiments, the characteristic peaks observed in XRD patterns and FTIR spectra remain nearly unchanged. This underscores the favorable stability of both chemical structures and crystalline structures for MP10DWP during the heating–cooling cycles (Fig. 6e, f). As a result, the MPDWPs showcased outstanding thermal and chemical tolerance, preserving not only the original latent heat but also the fusion/freezing point following 200 heating-cooling cycles. These findings robustly suggest that these MPDWPs exhibit stability and reliability, making them well-suited for practical applications in reversible thermal energy storage.

### Flame-Retardant Performance of Balsa-Derived CPCMs

**Fig. 7:**
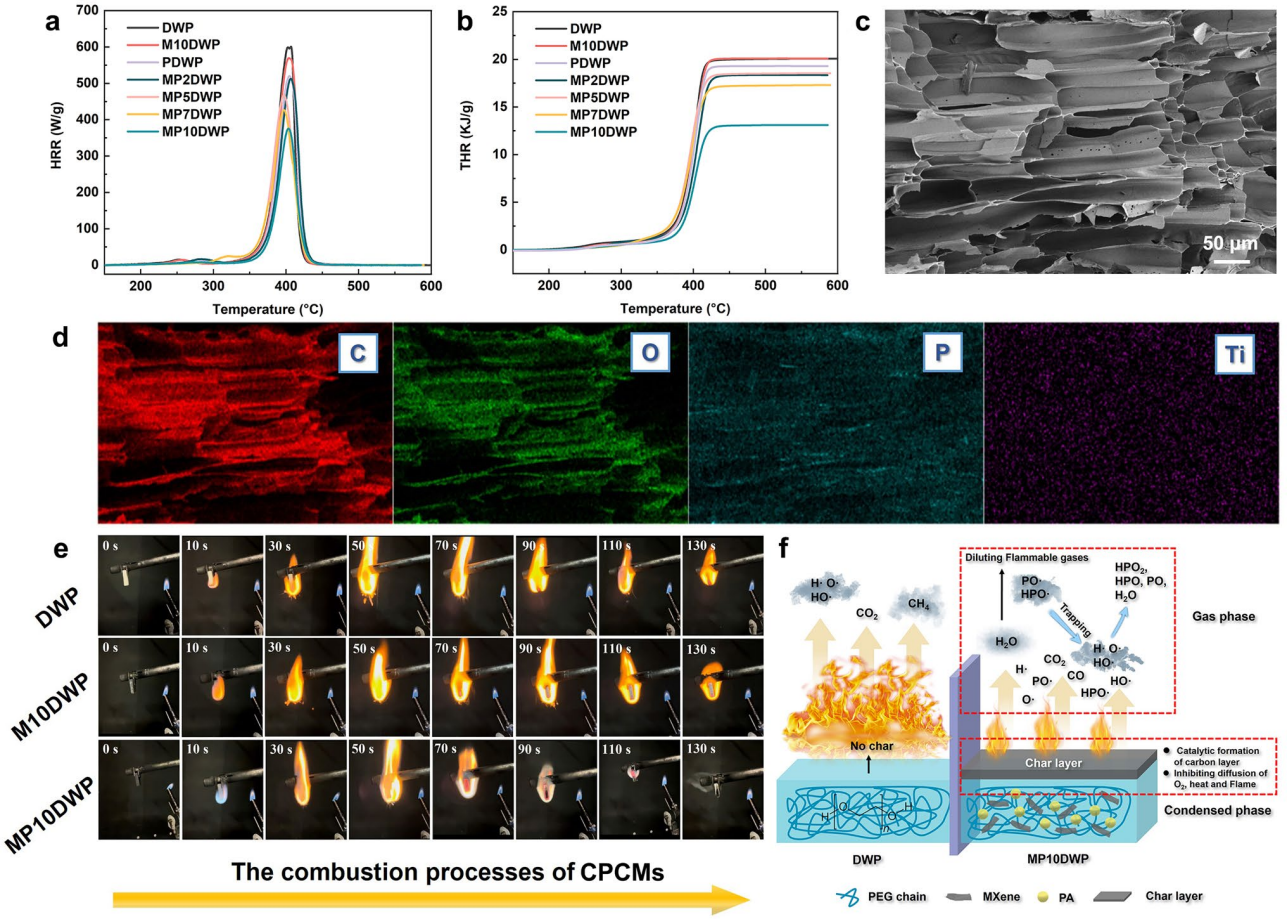
**a** HRR and **b** THR of DW, PEG, and MPDWPs. **c** SEM and d EDS mapping of char residue for MP10DWP. **e** Digital photographs of DWP and MPDWPs in the vertical burning test. **f** The possible fire-retardant mechanism of MPDWPs

The fire-retardant performance of CPCMs is crucial for ensuring safety during utilization. Given the size of CPCMs in this study, a micro combustion calorimeter (MCC) was employed to assess the combustion behavior. The results including heat release rate (HRR) and total heat release (THR) are presented in Fig. 7a, b, respectively. Also, corresponding data are recorded in Table S5. PEG encapsulated in unmodified nanowood, DWP, exhibits highly flammable characteristics, with peak heat release rate (pHRR) and THR of 600.12 W g^−1^ and 21.14 kJ g^−1^, respectively, which is attributed to the inherent flammability of both PEG and nanowood. Adding MXene alone (M10DWP) hardly has a significant impact on pHRR and THR. Even if the frame contains 10% MXene, the values of pHRR and THR are still as high as 569.44 W g^−1^ and 20.39 kJ g^−1^. Upon introducing MXene/PA hybrid structures, a decreasing trend is observed in both pHRR and THR for the CPCMs. Notably, with the addition of a hybrid structure only containing 2% MXene, there is a marginal change in MP2DWP, with the pHRR and THR decreasing by 512.53 W g^−1^, and 18.88 kJ g^−1^, respectively. However, as the MXene content increases, a more pronounced decrease in pHRR and THR is evident. Particularly, as for MP10DWP, this reduction reaches maximum, with a decrease of 375.44 W g^−1^ for pHRR and 13.47 kJ g^−1^ for THR. Additionally, an increase in the pHRR temperature is observed, rising from 382 to 395 °C. These observations suggest that the incorporation of MXene/PA hybrid structures can effectively retard the spread of flames, endowing the CPCMs with enhanced flame-retardant properties.

To further assess the flame-retardant performance of the CPCMs, a vertical burning test was conducted, and digital photographs from 0 to 130 s are presented in Fig. 7e. Clearly, DWP is prone to ignition, and the flame remains robust throughout the entire process, ultimately resulting in complete combustion. The only introduction of MXene structure did not impede the combustion behavior of the CPCMs. In the case of M10DWP, it displays nearly identical burning characteristics to DWP. With the addition of MXene/PA hybrid structures, the combustion behavior of MP10DWP is notably mitigated. It becomes challenging to ignite in the initial stages, and after 90 s, it initiates self-extinguishment while preserving the original appearance. These aforementioned observations are consistent with the results obtained from the MCC tests. Furthermore, the microscopic morphology of the residual char structure after CPCMs combustion was examined. DWP can undergo complete combustion, which exhibits disorderly fragmented char residuals without forming a dense structure (Fig. S3). As for MP10DWP, the honeycomb-like tracheid cell structure is remarkably preserved, suggesting the development of a resilient char during combustion (Fig. 7c). Notably, as shown in Fig. 7d, EDS mapping reveals a homogeneous distribution of phosphorus and titanium elements on the residual carbon of MP10DWP, with concentrations of 7.63% and 0.87%, respectively. These findings signify the creation of a stable porous residual char, achieved through the synergistic effect of PA and MXene, which effectively inhibit both combustion gas and heat transmission.

Thermogravimetry-Fourier transform infrared spectrum (TG-FTIR) analysis was employed to scrutinize the evolved gaseous species throughout the pyrolysis process, facilitating a more profound comprehension of the flame-retardant mechanism. As depicted in Fig. S4, the thermal decomposition process manifests FTIR characteristic peaks at 2930, 2350, and 1740 cm^−1^ within the temperature range of 200 to 450 °C. These peaks, corresponding to hydrocarbons, CO_2_/CO, and carbonyl compounds, respectively, denote the predominant thermal degradation events of the CPCMs in this temperature interval, which is consistent with the findings from the TG analysis in Sect. 3.4. Obviously, for MP10DWP, comparable temperature intervals for pyrolysis and analogous pyrolysis products are discernible in both two-dimensional and three-dimensional FTIR spectra (Fig. S5). This observation suggests that the incorporation of MXene/PA hybrid structures does not exert an influence on the pyrolysis pathways to the CPCMs, encompassing both the cellulosic framework and PEG molecular chains. However, in Fig. S4b, a new peak around 970 cm^−1^ assigned to the P‒O group emerges in MP10DWP [68]. This signifies the presence of phosphorus-containing groups originating from the MXene/PA hybrid structure during pyrolysis could possess the remarkable ability to scavenge highly reactive H• and HO• radicals, consequently playing a pivotal role in inhibiting combustion within the gaseous phases [69]. Further examination (Fig. S6) reveals that the introduction of MXene/PA hybrid structure diminishes the release of total pyrolysis products such as hydrocarbons, CO_2_, and carbonyl compounds, suggesting a restriction on the pyrolysis of both cellulosic component and PEG molecular chains. Consequently, it can be hypothesized that the release of phosphorus-containing groups promotes flame retardancy through the free radical quenching effect. Meanwhile, the inclusion of MXene/PA structure contributes to impeding the generation of flammable components through a barrier effect.

Drawing insights from the aforementioned analysis including residual char microscopic morphology and TG-FTIR examinations, an elucidated mechanism for the flame-retardant behavior of MPDWPs, that is PEG encapsulated in MXene/PA hybrid-modified nanowood, is postulated in Fig. 7f. During combustion or elevated temperature exposure, the MPDWPs undergo a decomposition process, liberating nonflammable gases, including CO_2_, H_2_O, and phosphorus-containing groups. These noncombustible gases serve dual roles by absorbing heat and concurrently diluting flammable gases and O_2_ within the surrounding atmosphere [70]. In addition, phosphorus-containing groups (*e.g.,* PO•, and HPO•) could trap the highly reactive H• and HO• radicals to transform the nonflammable H_2_O and potential phosphorus derivatives (*e.g.,* HPO_2_, HPO_3_, and PO_3_) [71]. Furthermore, this orchestrated modification is instrumental in the development of protective layers, stemming from the synergistic interaction between MXene and PA. The ensuing catalytic formation of a compact and continuous carbon layer is pivotal in curtailing smoke release and CO production, thereby augmenting the thermal decomposition stability and fire safety of the MPDWPs. Hence, we can hypothesize that the interplay of decomposed gases with phosphorus-containing groups and the formation of protective char layers catalyzed by MXene nanosheet synergistically contributes to the fire-retardant performance of wood-derived composite PCMs.

### Solar to Electricity Conversion of Balsa-Derived CPCMs

**Fig. 8:**
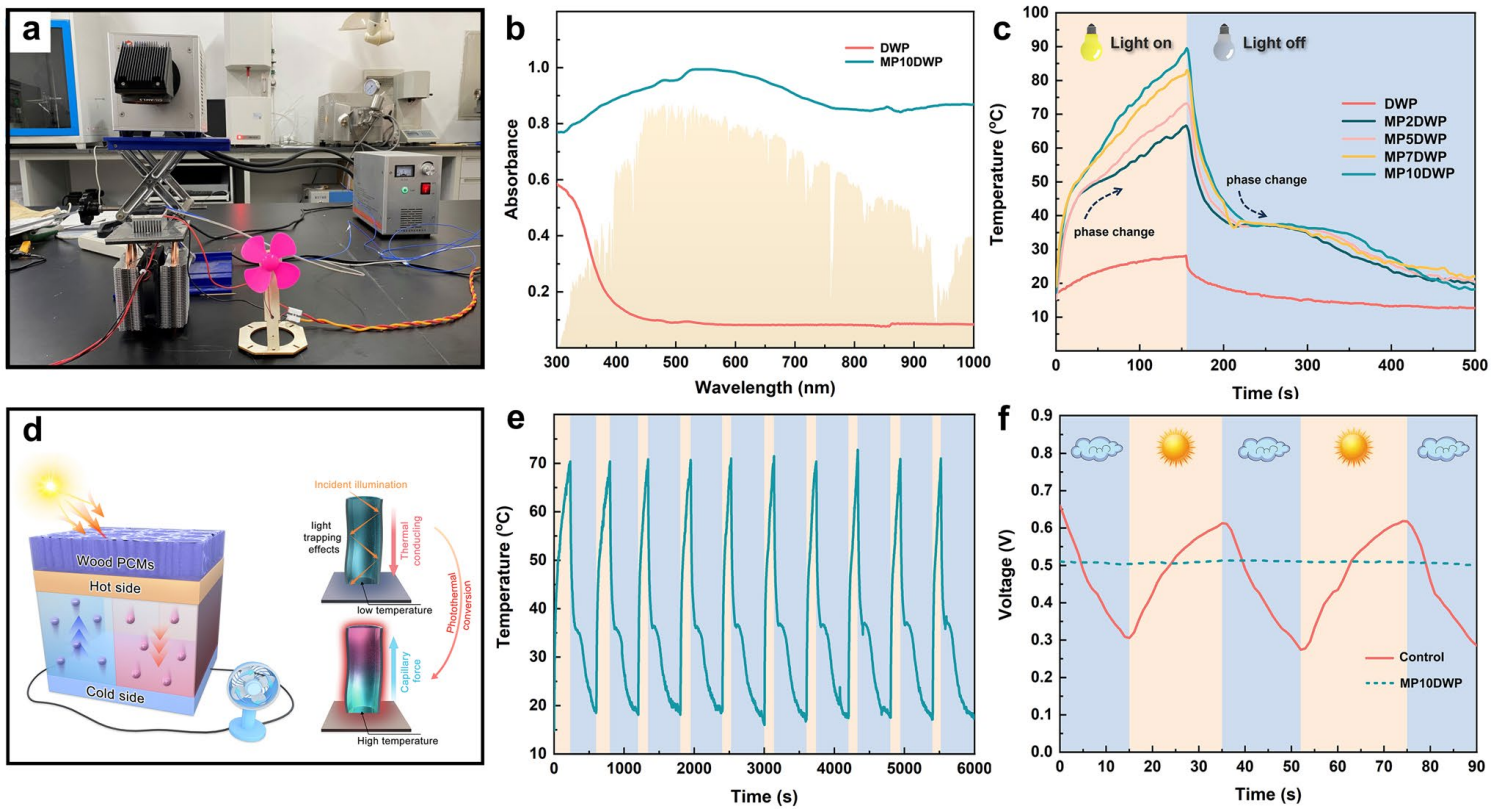
**a** Digital photograph of a testing system for solar to electricity conversion. **b** UV-Vis absorbance spectra of DWP and MP10DWP. **c** Temperature variation curves of DWP and MPDWPs during alternating Xenon lamp on and off. **d** Mechanism of hybrid-modified wood-encapsulated PCMs enabling effectively solar to electricity conversion. **e** 10 cycles of solar to thermal storage and release in MP10DWP during alternating Xenon lamp on and off. **f** Voltage fluctuations in a simulated cloudy weather scenario for testing systems with and without the application of MP10DWP

The core of the solar-thermal-electricity conversion system lies in the PCM, as it facilitates solar thermal energy storage and enables isothermal heat release during the phase change process, which is advantageous for ensuring the stable and continuous operation of the connected Seebeck thermoelectric elements. The experimental configuration designed for evaluating the solar to electricity conversion performance is depicted in Fig. 8a. Initially, we conducted examinations on the solar light absorption performance of DWP and MP10DWP using a UV-Vis spectrometer, and the results are illustrated in Fig. 8b. It is evident that DWP exhibits a weak absorption peak around 300 cm^−1^, possibly arising from residual lignin components in nanowood scaffolds. Meanwhile, DWP consistently exhibits a low absorbance below 0.1 with the wavelength range from 400 to 1000 cm^−1^, which indicates the subpar photothermal conversion performance of nanowood-encapsulated PCMs. However, as for MP10DWP, it demonstrates notably high absorbance throughout the entire detection spectrum, and even under visible light irradiation (390 to 790 cm^−1^), the absorbance value approaches 1. This observation can be attributed to the exceptionally high light absorption coefficient induced by the as-introduced MXene nanosheets, leading to an extraordinary level of light absorption ability within the MPDWPs.

Subsequently, the photothermal conversion behavior of DWP and MPDWPs was scrutinized under a Xenon lamp with an intensity of 1000 W m^−2^, equivalent to one solar irradiation (AM1.5G). As depicted in Figs. 8c and S7, the temperature of DWP failed to surpass 30 °C after 160 s of irradiation, remaining below the phase change threshold (*T*_*m*_). This observation highlights the insufficient solar to thermal conversion performance of CPCMs containing PEG and unmodified nanowood scaffolds. However, MP2DWP that includes a mere 2% mass fraction of MXene in nanowood scaffold has undergone a complete phase change thermal storage process with a peak temperature of up to 63.2 °C during the exposure to Xenon lamp sustaining 160 s, which suggests that a minimal quantity of MXene nanosheet had a pronounced impact on enhancing the light absorption capacity of the CPCMs, thereby positively contributing to the auxiliary improvement of solar to thermal conversion capacity. As expected, an increased content of the introducing MXene to the nanowood scaffold surface led to a gradual rise in the peak temperature under the same irradiation time, 160 s. Notably, when the additive content reaches 10%, MP10DWP attains a maximum temperature of 91.4 °C, making it own the outstanding potential for utilization in low-temperature solar thermal energy and conversion. Upon extinguishing the xenon lamp, DWP could hardly undergo a distinct phase change plateau and slowly return to room temperature solely through sensible heat release. In contrast, without the light irradiation of 340 s, MPDWPs commenced heat dissipation due to their higher thermal conductivity, nearly reaching the phase change temperature simultaneously, and then, they underwent an isothermal heat release process and returned to room temperature. Additionally, the solar to thermal conversion and storage efficiency (*ε*) is calculated based on Eq. (2) to further evaluate the photothermal behavior of MPDWPs [72].

2 $$\varepsilon = \frac{{m \times \Delta \,H_{m} }}{{P \times S \times \left( {t_{1} - t_{2} } \right)}} \times 100\%$$

where *m* represents the mass of the samples, Δ*H*_*m*_ denotes the phase change enthalpy, *P* signifies the light intensity, and *S* stands for the surface area of the samples. The parameters *t*_1_ and *t*_2_ correspond to the initial and final times of the melting process, respectively. It is evident that the *ε* value reaches an impressive 98.58%, showcasing a high level within existing solar thermal conversion and storage materials. As illustrated in Fig. 8d, it can be hypothesized that the remarkable photothermal conversion performance is attributed to the synergistic effect with both distinctive directional channels derived from nanowood and MXene surface deposition. On the one hand, the removal of lignin resulted in the thinning of wood cell wall, which could diminish the sunlight reflection. On the other hand, the numerous refractions occurring as a result of abundant sunlight entering the channels allow the deposited MXene to efficiently absorb and convert sunlight into thermal energy and rapidly store in the encapsulated PEG due to the improved thermal conductivity. Furthermore, following 10 cycles of heating and cooling, MP10DWP demonstrated consistent and repeatable thermal energy storage as well as heat release performance (Fig. 8e). Leveraging the exceptional photothermal conversion performance of the aforementioned MPDWPs, the solar-thermalelectricity conversion system with just four MP10DWP samples can consistently provide a voltage exceeding 0.5 V, ensuring the smooth operation of a small fan. Moreover, we simulated the cloudy conditions by covering the light source with a foam. As shown in Fig. 8f and Videos S2 and S3, without the addition of MPDWPs and only the presence of the photothermal conversion coating, the voltage fluctuates when simulated cloud cover intervenes. However, when the MP10DWP is present, a stable voltage output is maintained throughout the entire shading phase. Consequently, the designed wood functional modification strategy ensures an enhanced capacity for heat absorption and release of the as-encapsulated PCMs. This approach also opens avenues for efficient solar-thermal-electricity conversion in photoelectronic devices, ultimately contributing to the efficient utilization of solar resources.

### Electromagnetic Shielding Performance of Balsa-Derived CPCMs

**Fig. 9:**
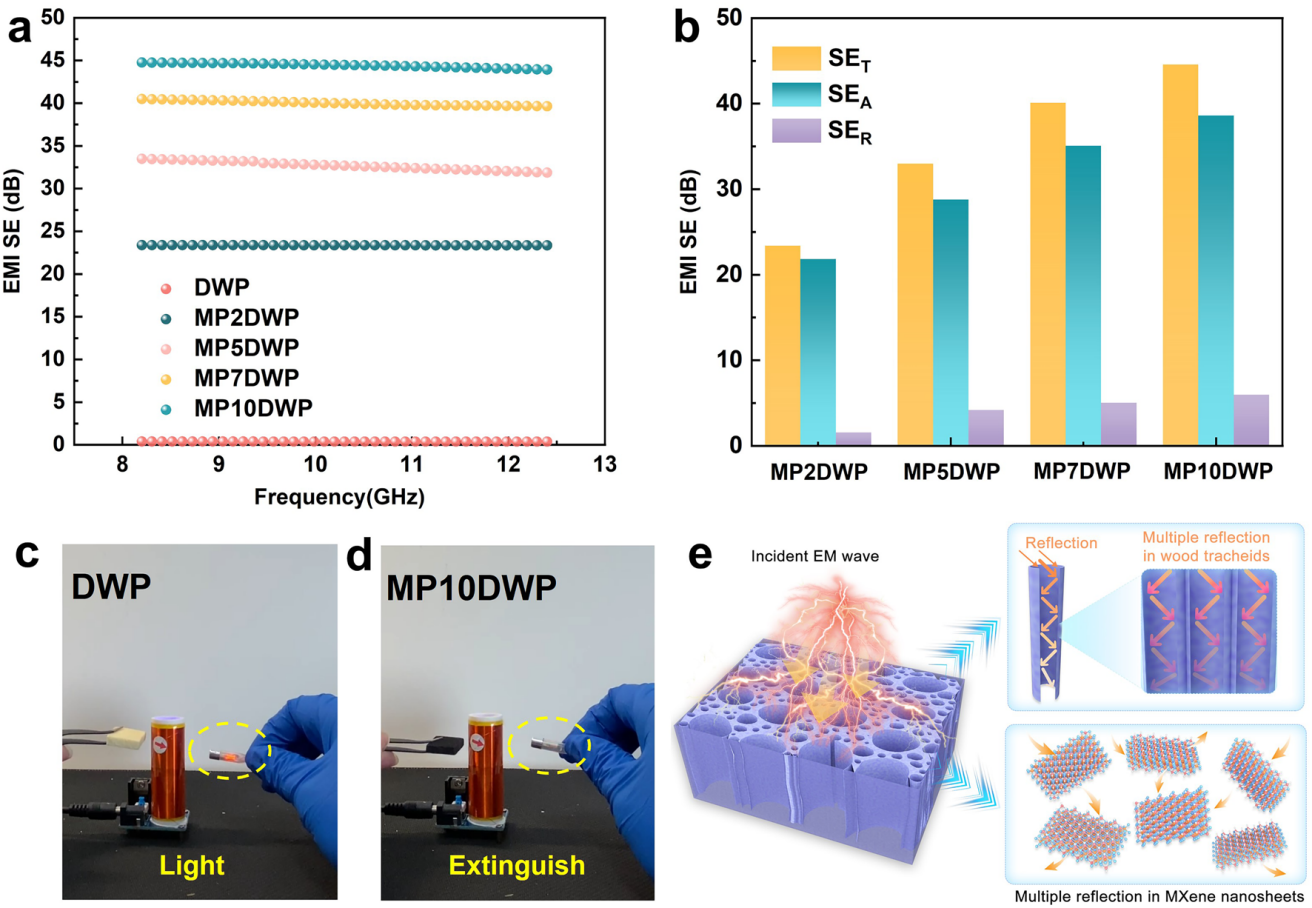
**a** Effect on EMI shielding effectiveness of DWP and MPDWPS with different MXene contents at the X-band. **b** Comparison of total EMI SE (SE_T_), microwave absorption (SE_A_), and microwave reflection (SE_R_) related to MPDWPs at 12.4 GHz frequency. **c, d** Digital photographs of EMI shielding tests of DWP and MP10DWP via Tesla coil. **e** Schematic of the EMI shielding of the MPDWPs

Given the contemporary health hazards associated with electromagnetic waves, imparting electromagnetic shielding performance to CPCMs holds significant importance for expanding their applications, particularly in wearable flexible devices and other fields [73]. The CPCMs including DWP and MPDWPs with the specified dimensions were tested for their shielding performance in the X-band (8.2–12.4 GHz) via a microwave vector network analyzer. In Fig. 9a, it is evident that the electromagnetic interference shielding effectiveness (EMI SE) of DWP, without the addition of MXene nanosheets, remained negligible within the whole tested frequency range. In contrast, MPDWPs exhibited a progressive enhancement in EMI shielding effects with an increasing deposition content of MXene on the nanowood scaffold surface. Remarkably, MP10DWP demonstrated a notable average EMI shielding value of 44.45 dB, which exceeds the commercial requirements (above 20 dB) for electromagnetic interference shielding products. In addition, as shown in Fig. 9c, d and the accompanying Videos S4 and S5, we conducted an experiment using a Tesla coil to validate the electromagnetic shielding performance. In the presence of an active Tesla coil emitting electromagnetic waves to illuminate an electromagnetic stimulated bulb, the brightness remained unbothered when DWP was introduced, indicating minimal electromagnetic shielding effectiveness, consistent with the results in Fig. 9a. As anticipated, when MP10DWP approached the coil, the bulb was observed to extinguish. This occurred because the intervention of MP10DWP disrupted the original propagation of electromagnetic waves, emphasizing the effectiveness of the designed MXene modification strategy.

We further meticulously calculated the relevant parameters including total EMI SE (SE_T_), microwave absorption (SE_A_), and microwave reflection (SE_R_) at a frequency of 12.4 GHz to gain a more profound understanding of the electromagnetic shielding mechanism in MPDWPs, as illustrated in Fig. 9b. For MP10DWP, SE_A_ with the value of 38.62 dB constituted a substantial 87.9% of the SE_T_ (43.94 dB), indicating that dominantly absorption electromagnetic interference (EMI) shielding mechanism prevails within the MPDWPs. The relatively smaller SE_R_ of all MPDWPs with a value lower than 6 dB indicates that incident electromagnetic waves likely penetrate the internal space of the composite with minimal reflection [74]. Thus, as shown in Fig. 9e, we can conjecture that the aforementioned exceptional electromagnetic shielding performance of MPDWPs is attributed to the exclusive unidirectional cellular encapsulation structure derived from the natural wood, coupled with the synergistic effect of stacked MXene nanosheet on wood cell wall surface, which offers moderate electrical conductivity and effective impedance matching, curbing significant reflection, and mitigating the risk of secondary pollution [75–77]. The wood-derived hierarchically cellular structure facilitates the repeated scattering and reflection of entered electromagnetic waves within the channels, which reinforces the interaction between the waves and internal interfaces, resulting in a multi-reflection effect. This effect ensures the continuous attenuation of penetrating waves, contributing to the improvement of SE_A_ in MPDWPs. Moreover, the EMI SE performance of the MP10DWP in our study surpasses that of many other wood-based materials (Table S6), underscoring the application of CPCMs for solar energy storage and conversion in correlation to electromagnetic shielding.

## Conclusions

In this work, we have innovatively crafted a type of wood-based composite phase change materials with multifunctional properties including highly efficient solar to thermal storage and conversion, excellent electromagnetic interference shielding, and robust flame retardancy. The synthesis process encompassed a facile and environmentally friendly approach, namely wood delignification followed by MXene/PA co-decoration, leveraging the inherent anisotropy and versatility of wood aerogel to support PEG. The wood-derived microporous structures as well as hydrophilic groups in the MXene/PA hybrid structure played a pivotal role in containing the leakage of PEG, capitalizing on robust surface tension, capillary forces, and hydrogen bonds. The as-prepared CPCMs showcase remarkable PEG packing yield and high thermal energy storage density, with thermal durability and stability throughout at least 200 heating and cooling cycles. In addition, the strategic deposition of MXene nanosheets on wood aerogel surfaces significantly enhanced both solar-thermal conversion efficiency (up to 98.58%) and electromagnetic interference shielding effectiveness with a maximum of 44.45 dB at the X-band. Furthermore, the addition of PA in conjunction with MXene also significantly restricts the flammability of the as-prepared CPCMs. The phenomenon of self-extinguishment is observed, particularly on MP10DWP, with critical fire-retardant parameters such as peak heat release rate and total heat release decreasing by 37.43% and 36.28%, respectively. Given these notable advantages, the multi-faceted approach, integrating facile MXene and PA hybrid wood modification, enhances the multifunctionality of the resulting form-stable composite phase change materials, contributing to extending the potential applications concerning solar energy harvesting.

## Supplementary Information

Below is the link to the electronic supplementary material.

Supplementary file1 (DOCX 1330 KB)

Supplementary file2 (MP4 4069 KB)

Supplementary file3 (MP4 3297 KB)

Supplementary file4 (MP4 2724 KB)

Supplementary file5 (MP4 2064 KB)
