# Supplementary material for: Leakage Proof, Flame-Retardant, and Electromagnetic Shield Wood Morphology Genetic Composite Phase Change Materials for Solar Thermal Energy Harvesting
Source: Nanomicro Lett. 2024 May 16;16:196. doi: 10.1007/s40820-024-01414-4 (PMC11099002; doi:10.1007/s40820-024-01414-4)
Supplement: Supplementary file 1 — Supplementary file1 (DOCX 1330 KB) [file 40820_2024_1414_MOESM1_ESM.docx]

Supporting Information for

**Leakage Proof, Flame-Retardant and Electromagnetic Shield Wood Morphology Genetic Composite Phase Change Materials for Solar Thermal Energy Harvesting**

Yuhui Chen^1^, Yang Meng^1,^ *, Jiangyu Zhang^1^, Yuhui Xie^1^, Hua Guo^3^, Mukun He^3^, Xuetao Shi^3^, Yi Mei^1^, Xinxin Sheng^2,^ *, and Delong Xie^1,^ *

^1^Yunnan Provincial Key Laboratory of Energy Saving in Phosphorus Chemical Engineering and New Phosphorus Materials, The International Joint Laboratory for Sustainable Polymers of Yunnan Province, The Higher Educational Key Laboratory for Phosphorus Chemical Engineering of Yunnan Province, Faculty of Chemical Engineering, Kunming University of Science and Technology, Kunming 650500, P. R. China

^2^Guangdong Provincial Key Laboratory of Functional Soft Condensed Matter, School of Materials and Energy, Guangdong University of Technology, Guangzhou 510006, P. R. China

^3^Shaanxi Key Laboratory of Macromolecular Science and Technology, School of Chemistry and Chemical Engineering, Northwestern Polytechnical University, Xi’an, Shaanxi 710072, P. R. China

*Corresponding authors. E-mail: [mengyang@kust.edu.cn](mailto:mengyang@kust.edu.cn) (Yang Meng), [xinxin.sheng@gdut.edu.cn](mailto:xinxin.sheng@gdut.edu.cn) (Xinxin Sheng), [cedlxie@kust.edu.cn](mailto:cedlxie@kust.edu.cn) (Delong Xie)

**S1 Experimental Sections**

**S1.1 Materials**

Wood resources, balsa timbers, with dimensions of 10 mm×10 mm×1000 mm were purchased from the JIMUM online shop on the Tmall website, China. MXene precursor, Ti_3_AlC_2_ MAX phase with 300 mesh and 99.9% purity, was provided by XFNANO Co. Ltd., Nanjing, China. Other main chemical reagents such as sodium chlorite (NaClO_2_, AR), glacial acetic acid (CH_3_COOH, AR), poly (ethylene glycol) (PEG, Mn =2000 g/mol), lithium fluoride (LiF, 99.9 % metals basis), hydrochloric acid (HCl aqueous solution, 1 mol/L), sodium hydroxide (NaOH, AR), and phytic acid (PA, 50 wt%) were all supplied by Shanghai Aladdin Biochemical Technology Co., Ltd., China. In addition, deionized water was provided by our laboratory.

**S1.2 Synthesis of MXene (Ti_3_C_2_Tx) from MAX (Ti_3_AlC_2_) phase**

MXene (Ti_3_C_2_Tx) nanosheets were synthesized using a refined etching method involving LiF and HCl [1, 2], with nuanced adjustments for optimization. In this method, 2 g of MAX (Ti_3_AlC_2_) was slowly introduced into an aqueous solution containing 2 g of LiF and 9 M HCl. The etching process was meticulously sustained at 35 °C for a duration of 24 h. Subsequently, the obtained sample underwent centrifugation at 3500 rpm for 5 min, followed by thorough washing with deionized water until achieving a pH near 6. The material was then re-dispersed in DI water and subjected to ultrasonication for 20 min at 0 °C in an ice bath. Post-ultrasonication, the dark-green supernatant was collected after centrifugation at 3500 rpm for 20 min, resulting in a well-dispersed suspension of delaminated MXene nanosheets with a concentration of 5.2 mg/mL.

**S1.3 Characterization**

The chemical composition analysis of all samples employed Fourier transform infrared spectroscopy (FTIR, ATR mode) within the 400 - 4000 cm^-1^ range, with a resolution of 4 cm^-1^. Morphological characteristics were examined using field emission scanning electron microscopy (SEM, Zeiss Sigma) at an accelerating voltage of 10 kV. X-ray diffraction (XRD) patterns were acquired in the 2θ range of 3°- 80° using CoKα radiation on a Bruker D8 Advance Bragg-Brentano diffractometer. The primary components in balsa wood were identified following the NREL/TP-510-42,618 analytical method from the National Renewable Energy Laboratory (NREL). Acid-insoluble lignin content was verified through sulfuric acid (72 wt%) hydrolysis, while hemicellulose and α-cellulose content were calculated based on sugar analyses utilizing high-performance anion exchange chromatography (Dionex 3000). Thermal properties of pure PEG and balsa-based FPCMs, both pre-and post-200 heating/cooling cycles, were investigated using a differential scanning calorimeter (DSC) operating from 0 to 70 °C (5 °C/min rate) under a nitrogen atmosphere. Thermal conductivity was measured with a C-Therm TCi™ Thermal Conductivity Analyzer. Specific heat capacity was determined using a TA instrument (DSC Q1000) with sapphire calibration, and the thermal conductivity was reported at a temperature of 25 °C. Thermal stability and degradation were studied through thermogravimetric analysis on a TA instrument (TGA Q5500). The samples were heated from 30 to 800 °C at a rate of 10 °C/min under a nitrogen gas flow of 60 mL/min. The flammability properties of the CPCMs were evaluated using the Fire Testing Technology (FTT) microscale combustion colorimeter (MCC, ASTM E1354-17, U.K.). Fourier transform infrared−thermogravimetric analysis (FTIR-TGA) spectra were recorded using a Bruker TGA-IR between 30 and 600 °C in a nitrogen atmosphere.

**S1.4 Solar to electricity conversion test**

The solar-thermal-electric conversion test was conducted on a homemade testing system, which includes a xenon lamp light source, a refrigeration unit, Seebeck elements, a multimeter combined with PT100 temperature probes and wires to record temperature and voltage changes, and a small electric fan. The specific steps are as follows:

1. Place the composite material on the surface of the Seebeck elements, bonded in the middle with thermally conductive silicone gel.
2. Turn on the refrigeration unit to maintain the temperature at the cold end at 0℃.
3. Then, turn on the xenon lamp and adjust the light intensity to reach a solar intensity of AM1.5, 1000W/m**^2^**. Record the temperature changes on the surfaces of different composite materials during solar irradiation and the voltage generated by Seebeck using the multimeter.
4. Simultaneously, after 10 cycles of turning the xenon lamp on and off, the stability of heat management and conversion is also tested.

**S1.5 Solar to electricity conversion test**

Electromagnetic shielding measurements were performed using an Agilent PNA-N5244A vector network analyzer employing a waveguide method within the frequency range of 8.2 - 12.4 GHz. The dimensions of the tested sample were 20 mm × 10 mm × 3 mm. Scatter parameters (*S*_11_ and *S*_21_) were recorded to derive the coefficients of reflection (R), absorption (A), and transmission (T). The total electromagnetic interference shielding effectiveness (EMI *SE*_T_) of the MPDWPs was calculated using the following formula:

| $R=\left\vert S_{11} \right\vert^{2}$ | (S1) |
| --- | --- |
| $T=\left\vert S_{21} \right\vert^{2}$ | (S2) |
| $A=1-R-T$ | (S3) |
| ${SE}_{T}={SE}_{A}+{SE}_{R}$ | (S4) |
| ${SE}_{A}=-10\log\left( \frac{T}{1-R} \right)$ | (S5) |
| ${SE}_{R}=-10\log\left( 1-R \right)$ | (S6) |
| ${SE}_{T}=-10\log\left( T \right)$ | (S7) |

Where, *SE*_A_, and *SE*_R_ represent microwave adsorption, and microwave reflection, respectively.

**S2 Supplementary Tables**

**Table S1** Chemical composition, density, and porosity of raw balsa wood (RW) and delignified balsa wood (DW)

| Samples | Cellulose  (%) | Hemicellulose  (%) | Lignin  (%) | Density  (kg/m^3^) | Porosity  (%) |
| --- | --- | --- | --- | --- | --- |
| RW | 43.1±3.2 | 31.7±1.6 | 25.2±3.4 | 74.2±1.9 | 90.3±2.3 |
| DW | 73.2±2.9 | 22.9±3.3 | 3.9±0.9 | 44.5±1.1 | 97.4±1.1 |

Note: Lignin content was acid-insoluble lignin in wood determined according to the analytical method NREL/TP-510-42618. And hemicellulose and cellulose content in wood was determined by sugar analysis.

**Table S2** DSC data for pure PEG and wood-based CPCMs

| Samples | *T*_m_ (℃) | Δ*H*_m_ (kJ/kg) | *T*_c_ (℃) | Δ*H*_c_ (kJ/kg) | *I* (%) | *F* (%) | *E* |
| --- | --- | --- | --- | --- | --- | --- | --- |
| PEG | 47.7±0.5 | 155.9±4.5 | 36.4±0.4 | 152.3±3.9 | 100 | 100 | 1 |
| DWP | 48.9±0.3 | 145.7±3.1 | 36.0±0.4 | 142.0±3.6 | 95.5 | 93.5 | 0.98 |
| MP2DWP | 45.5±0.6 | 143.5±3.3 | 32.8±0.3 | 139.8±2.7 | 94.6 | 92.0 | 0.97 |
| MP5DWP | 46.8±0.4 | 141.7±4.7 | 32.2±0.4 | 137.6±2.5 | 93.4 | 90.9 | 0.97 |
| MP7DWP | 47.1±0.4 | 140.36±2.5 | 32.0±0.2 | 137.2±3.4 | 92.7 | 90.0 | 0.97 |
| MP10DWP | 49.2±0.4 | 135.5±2.9 | 33.8±0.3 | 131.8±2.7 | 91.5 | 86.9 | 0.95 |

Note: Ideal phase transition enthalpy (*I*) of the prepared CPCMs is calculated using PEG mass loading per unit mass (%). The expected value *E* (F/I), serves as a metric to gauge the degree of influence the encapsulation framework has on phase-change enthalpy values.

**Table S3** Comparison of thermal conductivity reported for related wood-based CPCMs

| Wood-based CPCMs | Thermal Conductivity (W/mK) | Refs |
| --- | --- | --- |
| Carbonized wood/ 1-tetradecanol | 0.669 | [S3] |
| Carbonized wood/ Mg(NO_3_)_2_·6H_2_O | 0.41 | [S4] |
| Wood/1-octadecene | 0.19 | [S5] |
| Wood/GMA modified PEG | 0.32 | [S6] |
| Wood/SiO_2_ modified PEG | 0.33 | [S7] |
| MD10DWP | 0.82 | This work |

**Table S4** Thermal gravimetric (TG) data of DW, PEG, and wood-based CPCMs

| Sample | *T*-5 wt% (℃) | *T*_max_ (℃) | *R*_max_ (%/min) | Char yield (wt%) |
| --- | --- | --- | --- | --- |
| DW | 248.9 | 323.6 | 37.66 | 28.1 |
| PEG | 340.4 | 402.6 | 22.2 | 2.7 |
| DWP | 256.0 | 335.0 | 21.4 | 39.3 |
| MP2DWP | 306.4 | 404.3 | 50.3 | 7.3 |
| MP5DWP | 282.9 | 399.8 | 39.1 | 8.2 |
| MP7DWP | 259.1 | 399.1 | 36.4 | 8.6 |
| MP10DWP | 290.8 | 401.4 | 39.7 | 10.1 |

**Table S5** Combustion performance parameters of DWP and MPDWPs

| Samples | pHRR (W/g) | λ_1_ (%) | THR (kJ/g) | λ_2_ (%) |
| --- | --- | --- | --- | --- |
| DWP | 600.12 | 0 | 21.14 | 0 |
| M10DWP | 569.44 | 5.09 | 20.39 | 3.54 |
| MP2DWP | 512.53 | 14.59 | 18.88 | 10.69 |
| MP5DWP | 466.50 | 22.26 | 15.76 | 25.45 |
| MP7DWP | 426.53 | 28.92 | 15.88 | 24.88 |
| MP10DWP | 375.44 | 37.43 | 13.47 | 36.28 |

**Table S6** Comparison of the EMI shielding value of MP10DWP and different composites in other references

| Composites | EMI SE (dB) | Refs |
| --- | --- | --- |
| Carbonized wood/paraffin CPCMs | 24.4 | [S8] |
| MXene modified wood | 32.7 | [S9] |
| ZIF-67/ Carbonized wood CPCMs | 44 | [S10] |
| Magnetic Wood | 10 | [S11] |
| Superhydrophobic MXene-modified wood | 43.4 | [S12] |
| MP10DWP | 44.45 | This work |

**S3 Supplementary Figures**


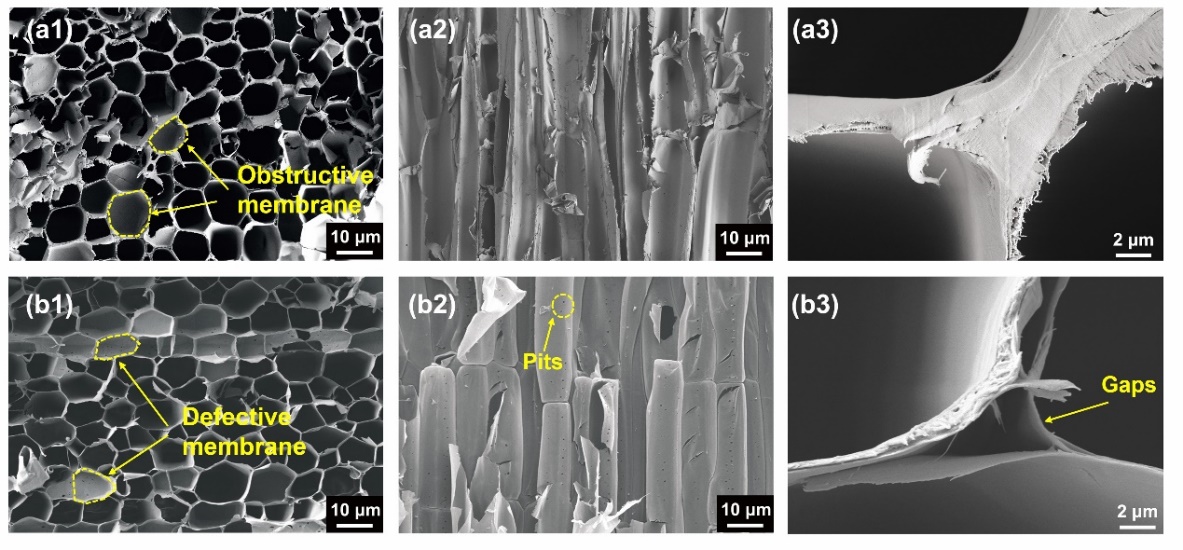


**Fig. S1** Field-emission scanning electron microscope of **(a1)** cross view, **(a2)** longitudinal view, and **(a3)** wood cell wall in cross view of raw balsa wood; (b1) cross view, **(b2)** longitudinal view, and **(b3)** wood cell wall in cross view of delignified wood.


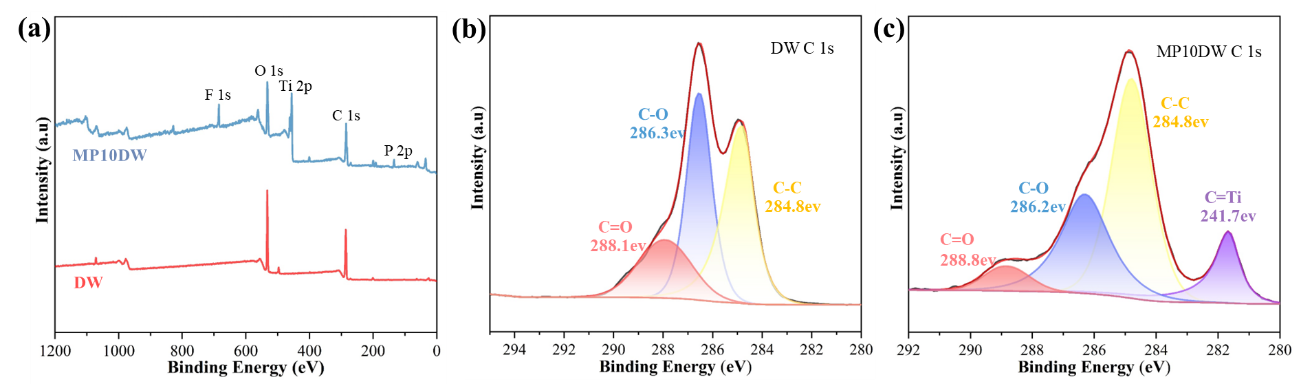


**Fig. S2 (a)** XPS spectra of DW and MP10DW, **(b)** high-resolution C 1s peaks of DW and **(c)** high-resolution C 1s peaks of MP10DW

Fig. S2a displays the complete XPS spectra of delignified wood (DW) and MP10DW. Compared to DW, MP10DW exhibits new characteristic peaks of F, Ti, and P at 685.3, 456.1, and 134.3 eV, respectively. Additionally, the presence of a new C-Ti bond in MP10DW further indicates the formation of new covalent bonds between the MXene/PA hybrid structure and DW (Fig. S2b, c). The difference in the binding energy of the carbon-oxygen double bond also suggests alterations in the chemical environment and hydrogen bonding interactions between the MXene/PA hybrid structure and cellulose.


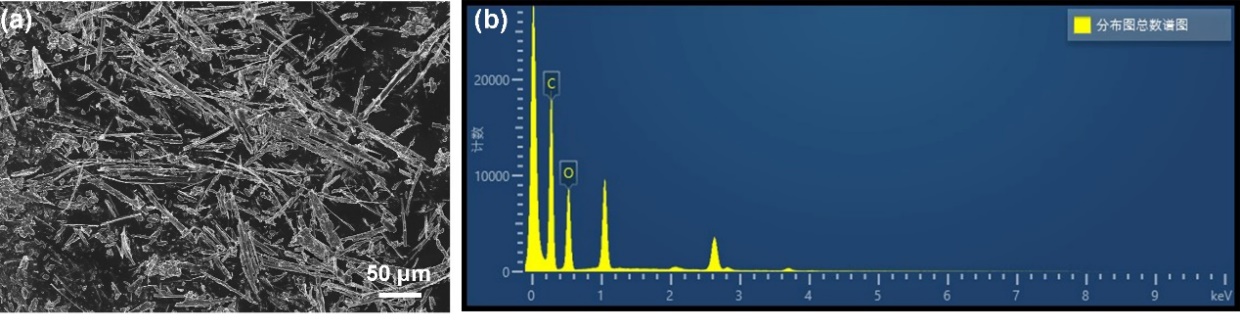


**Fig. S3 (a)** SEM and **(b)** EDS of char residue for DWP


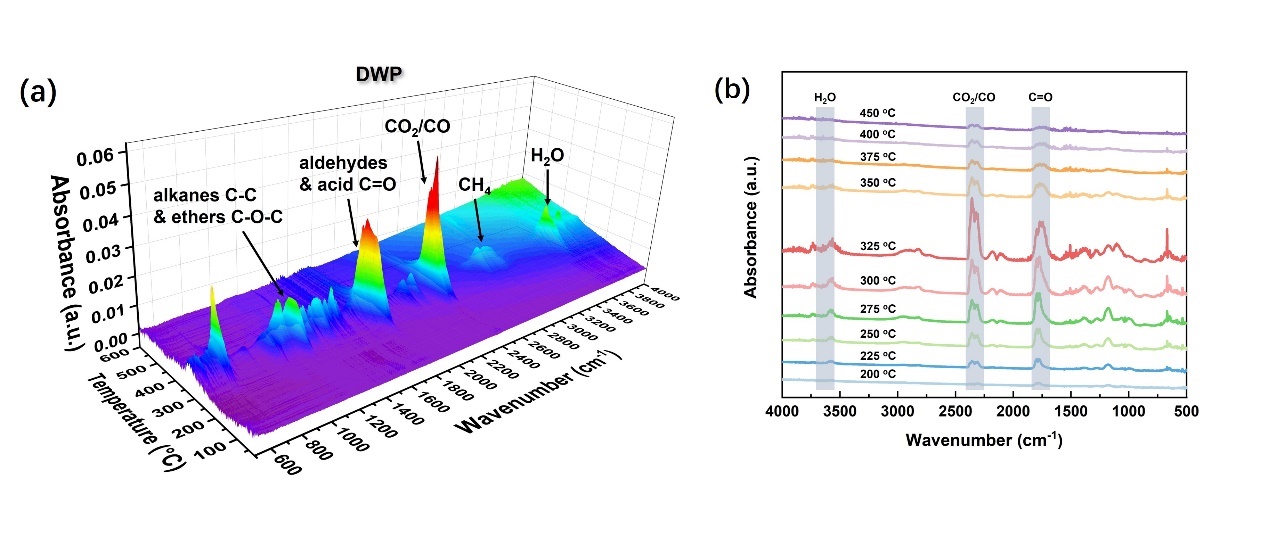


**Fig. S4** 3D TG-IR spectra of (**a**) DWP and (**b**) FTIR spectra of the pyrolysis products for DWP at different temperatures


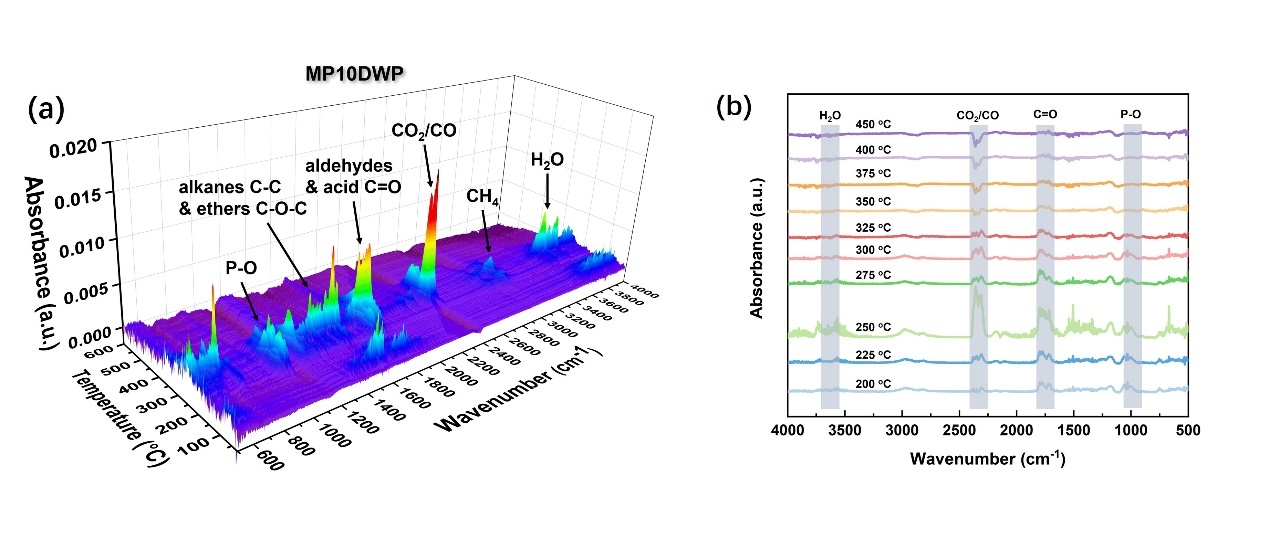


**Fig. S5** 3D TG-IR spectra of (**a**) MP10DWP and (**b**) FTIR spectra of the pyrolysis products for MP10DWP at different temperatures


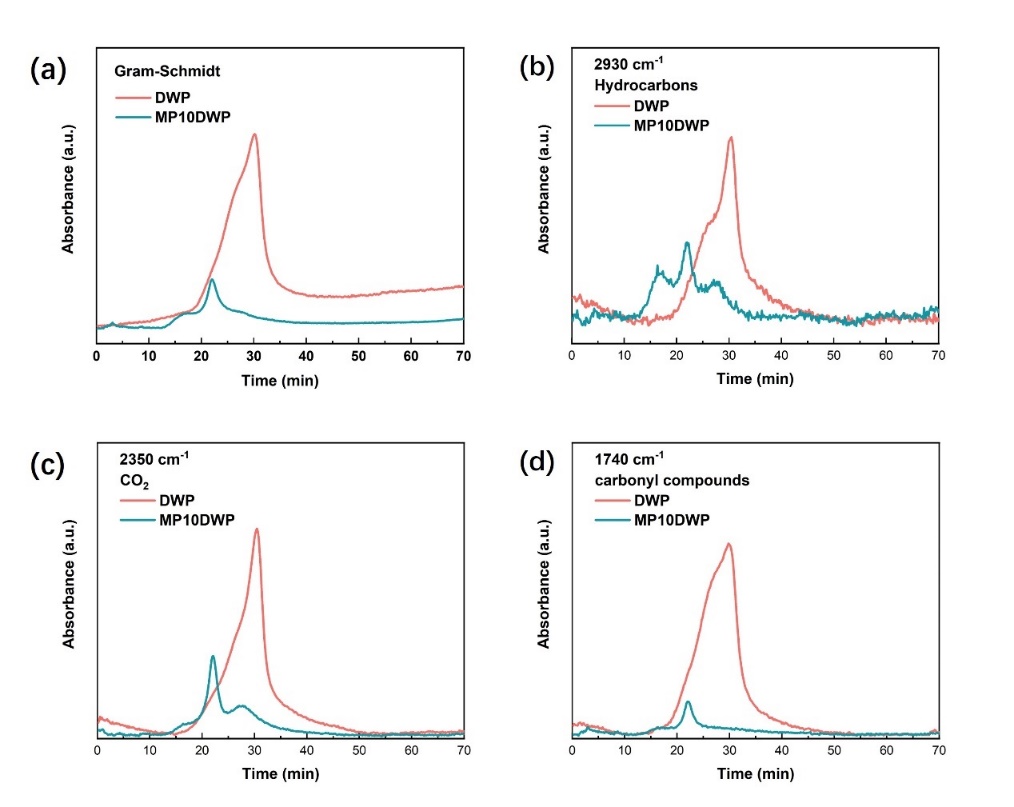


**Fig. S6** TG-IR results of DWP and MP10DWP: (**a**) total pyrolysis products, (**b**) hydrocarbons, (**c**) CO_2_, (**d**) carbonyl compounds


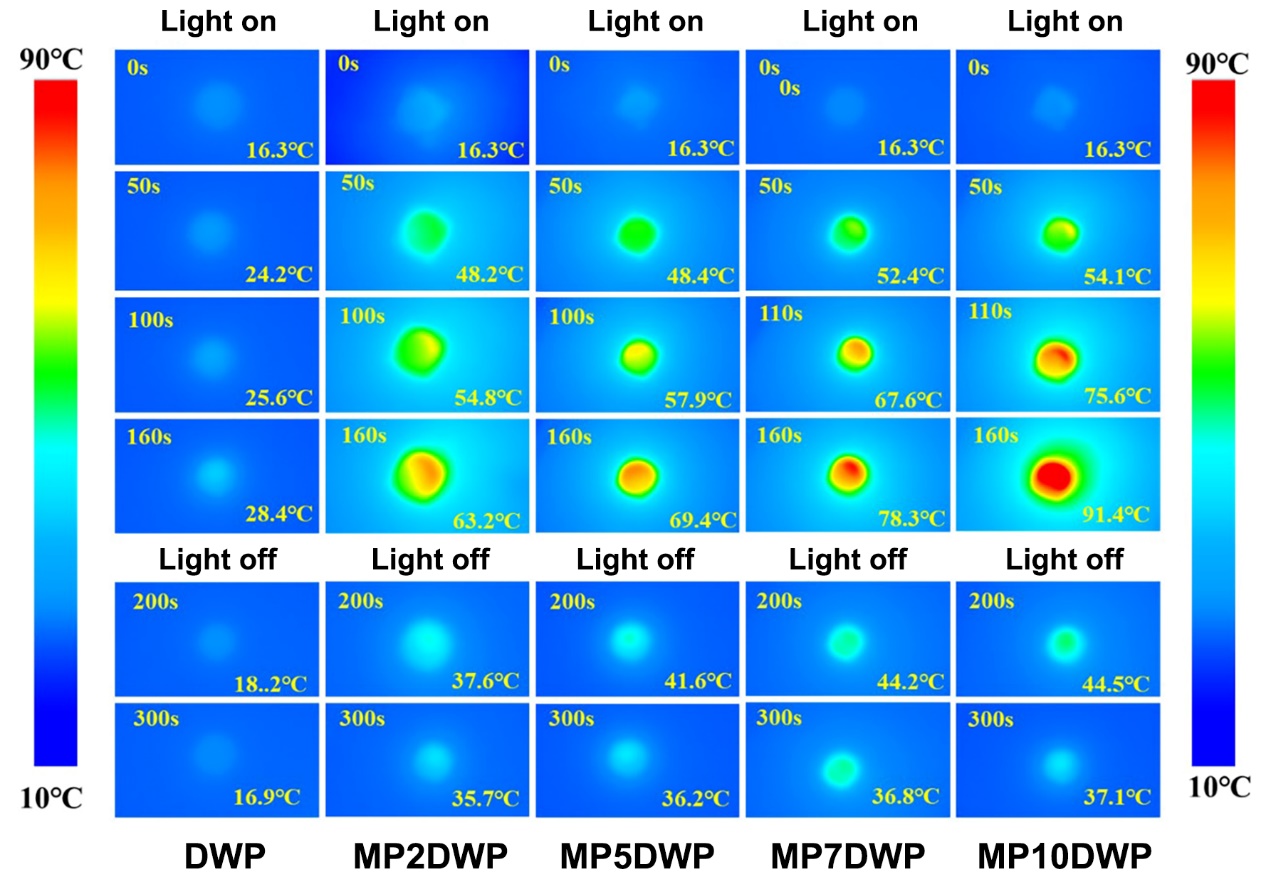


**Fig. S7** Infrared thermography of DWP and MPDWPs during alternating Xenon lamp on and off

**Supplementary References**

1. Y. Cao, M. Weng, M.H.H. Mahmoud, A.Y. Elnaggar, L. Zhang et al., Flame-retardant and leakage-proof phase change composites based on MXene/polyimide aerogels toward solar thermal energy harvesting. Adv. Compos. Hybrid Mater. **5**, 1253–1267 (2022). <https://doi.org/10.1007/s42114-022-00504-4>
2. H. Jiang, J. Li, Y. Xie, Y. Du, J. Zhao et al., Rapid exfoliation and surface hydroxylation of high-quality boron nitride nanosheets enabling waterborne polyurethane with high thermal conductivity and flame retardancy. Adv. Compos. Hybrid Mater. **7**, 8 (2024). <https://doi.org/10.1007/s42114-023-00818-x>
3. H. Yang, Y. Wang, Q. Yu, G. Cao, X. Sun et al., Low-cost, three-dimension, high thermal conductivity, carbonized wood-based composite phase change materials for thermal energy storage. Energy **159**, 929–936 (2018). <https://doi.org/10.1016/j.energy.2018.06.207>
4. H. Zhang, Z. Ling, S. Zhou, X. Fang, W. Zhang, Preparation and characteristic of wood-based inorganic composite phase change material with effective anisotropic thermal conductivity for thermal energy storage. Sol. Energy Mater. Sol. Cells **251**, 112172 (2023). <https://doi.org/10.1016/j.solmat.2022.112172>
5. Z. Qiu, S. Wang, Y. Wang, J. Li, Z. Xiao et al., Transparent wood with thermo-reversible optical properties based on phase-change material. Compos. Sci. Technol. 200 (2020) 108407. <https://doi.org/10.1016/j.compscitech.2020.108407>
6. Y. Li, B. Wang, W. Zhang, J. Zhao, X. Fang et al., Processing wood into a phase change material with high solar-thermal conversion efficiency by introducing stable polyethylene glycol-based energy storage polymer. Energy **254**, 124206 (2022). <https://doi.org/10.1016/j.energy.2022.124206>
7. J. Xu, T. Yang, X. Xu, X. Guo, J. Cao Processing solid wood into a composite phase change material for thermal energy storage by introducing silica-stabilized polyethylene glycol. Compos. Part A Appl. Sci. Manuf. **139**, 106098 (2020). <https://doi.org/10.1016/j.compositesa.2020.106098>
8. M. Zhou, J. Wang, Y. Zhao, G. Wang, W. Gu et al., Hierarchically porous wood-derived carbon scaffold embedded phase change materials for integrated thermal energy management, electromagnetic interference shielding and multifunctional application. Carbon **183**, 515–524 (2021). <https://doi.org/10.1016/j.carbon.2021.07.051>
9. Y. Jiang, X. Ru, W. Che, Z. Jiang, H. Chen et al., Flexible, mechanically robust and self-extinguishing MXene/wood composite for efficient electromagnetic interference shielding. Compos. Part B Eng. **229**, 109460 (2022). <https://doi.org/10.1016/j.compositesb.2021.109460>
10. S. Liu, B. Quan, M. Sheng, Y. Yang, X. Hu et al., A novel *in situ* growth ZIF-67 on biological porous carbon encapsulated phase change composites with electromagnetic interference shielding and multifunctional energy conversion. Nano Energy **114**, 108669 (2023). <https://doi.org/10.1016/j.nanoen.2023.108669>
11. Z. Cheng, Y. Wei, C. Liu, Y. Chen, Y. Ma et al., Lightweight and construable magnetic wood for electromagnetic interference shielding. Adv. Eng. Mater. **22**, 2000257 (2020). <https://doi.org/10.1002/adem.202000257>
12. X. Hao, L. Xing, D. Li, J. Wang, W. Lan et al., Superhydrophobic, mechanically robust and self-cleaning MXene/wood composite for high-performance electromagnetic interference shielding. Ind. Crops Prod. **199**, 116744 (2023). <https://doi.org/10.1016/j.indcrop.2023.116744>
